# Characterization of immune phenotypes in peripheral blood of adult renal transplant recipients using mass cytometry (CyTOF)

**DOI:** 10.1093/immhor/vlae013

**Published:** 2025-02-18

**Authors:** Sangeeta Kowli, Sheroy Minocherhomji, Olivia M Martinez, Stephan Busque, Herve Lebrec, Holden T Maecker

**Affiliations:** Institute for Immunity, Transplantation and Infection, School of Medicine, Stanford University, Stanford, CA, United States; Department of Microbiology and Immunology, School of Medicine, Stanford University, Stanford, CA, United States; Amgen Inc., Thousand Oaks, CA, United States; Eli Lilly and Company, Indianapolis, IN 46285, United States; Division of Abdominal Transplantation, Department of Surgery, Stanford University, Stanford, CA, United States; Division of Abdominal Transplantation, Department of Surgery, Stanford University, Stanford, CA, United States; Amgen Inc., Thousand Oaks, CA, United States; Sonoma Biotherapeutics, South San Francisco, CA 94080, United States; Institute for Immunity, Transplantation and Infection, School of Medicine, Stanford University, Stanford, CA, United States; Department of Microbiology and Immunology, School of Medicine, Stanford University, Stanford, CA, United States

**Keywords:** CyTOF, immune profiling, PBMC, renal transplant

## Abstract

Chronic immunosuppressive therapies are crucial in organ transplantation but can increase the risk of opportunistic infections and cancer over time. We investigated immune status changes in 10 kidney transplant patients and 11 age-matched healthy adults using broad in vitro stimulation of subject-derived peripheral blood mononuclear cells followed by mass cytometry by time of flight over 6 mo. Overall, the immune cells of transplant patients exhibited increased CD8^+^ T cell activation and differentiation compared with healthy donors, with elevated CD8^+^ CD57^+^, MIP-1β, and interferon γ production (*P < *0.05, *P* < 0.05, and *P < *0.01, respectively). CD107a and granzyme B expression were increased in CD8^+^ T cells and CD56^bright^ natural killer cells (*P < *0.05 and *P < *0.01, respectively), while T regulatory cells had decreased interleukin-10 production (*P < *0.05). These changes indicated a proinflammatory environment influenced by induction therapy and ongoing maintenance drugs. Additionally, transplant recipients displayed signs of immune modulation, including decreased tumor necrosis factor α, interferon γ, and MIP-1β expression in γδT cells (*P < *0.05 and *P < *0.01), and reduced interleukin-17 and granulocyte-macrophage colony-stimulating factor expression in CD8+ T memory cell subsets (*P < *0.05). The diverse functional changes underscore the importance of comprehensive immune status profiling for optimizing individual treatment strategies and developing better immunosuppressants that specifically target activated cell populations.

## Introduction

For patients with end-stage renal failure, kidney transplantation is the treatment of choice compared with dialysis, as patients have both a survival advantage and a better quality of life.^1–3^ Over the years, there has been tremendous progress in the field of solid organ transplantation, primarily in short-term allograft survival.^4^ However, despite advances including the development of newer immunosuppressants and improved medical care, the success of long-term graft survival is still unsatisfactory.^3–5^

Chronic immunosuppressive therapy is associated with graft toxicity, the risk of de novo cancers, infections, and cardiovascular disease, contributing to major complications and increased morbidity and mortality of transplant patients.^6^ To ensure a successful transplantation, it is essential to evaluate the immune health of the transplant patients both before and after the procedure. This evaluation would help in identifying immune alterations caused by underlying primary disease and the effects of immunosuppressants. Additionally, it would enable monitoring of risks associated with potential viral and bacterial infections, and the risks of developing cancers, including post-transplantation lymphoproliferative disorders. Comprehensive monitoring of the immune profile can also be leveraged for potential treatment adjustments.^6^^,^^7^

A major limitation in the field has been the lack of comprehensive follow-up monitoring of transplant patients that would help identify early immune perturbations that contribute to an altered immune system and chronic rejection of the graft.^8^ However, there is a poor understanding of which immune cell parameters should be measured to inform risk. The goal of this study was therefore to identify (1) differences in immune phenotypes and function between age-matched healthy individuals and transplant patients and (2) changes in immune phenotypes and function within transplant patients over time. To fulfill this goal, we used mass cytometry (cytometry by time of flight [CyTOF]), which can simultaneously detect ∼40 phenotypic and functional immune cell markers,^9^ to elucidate differences in cell frequencies, cytokine expression, activation, and cytotoxicity markers, providing an in-depth analysis of the effects of immunosuppressive therapy on the immune status of transplant recipients.

In this study, we performed high-dimensional analysis of unstimulated and stimulated peripheral blood cells from 10 adult renal transplant recipients (4 females, 6 males), prior to transplantation (baseline) and 1, 3, and 6 mo post-transplantation, comparing them with 11 age-matched healthy donors (7 females, 4 males). Further, to also identify the effects of immunosuppressive therapy on the transplant recipients, we investigated changes post-transplantation vs baseline values. Such information constitutes an important resource in understanding core immune changes associated with post-transplant immunosuppression. These studies lay the groundwork for making informed decisions to design and optimize personalized treatment strategies for transplant patients, including choosing between the use of standard combined immunosuppression, the use of monoimmunosuppressive therapy, or weaning of immunosuppression, to maintain optimal health of the transplant recipient and maximize the success of the transplant and patient survival.

## Materials and methods

### Patients and healthy control subjects

**Figure 1.**
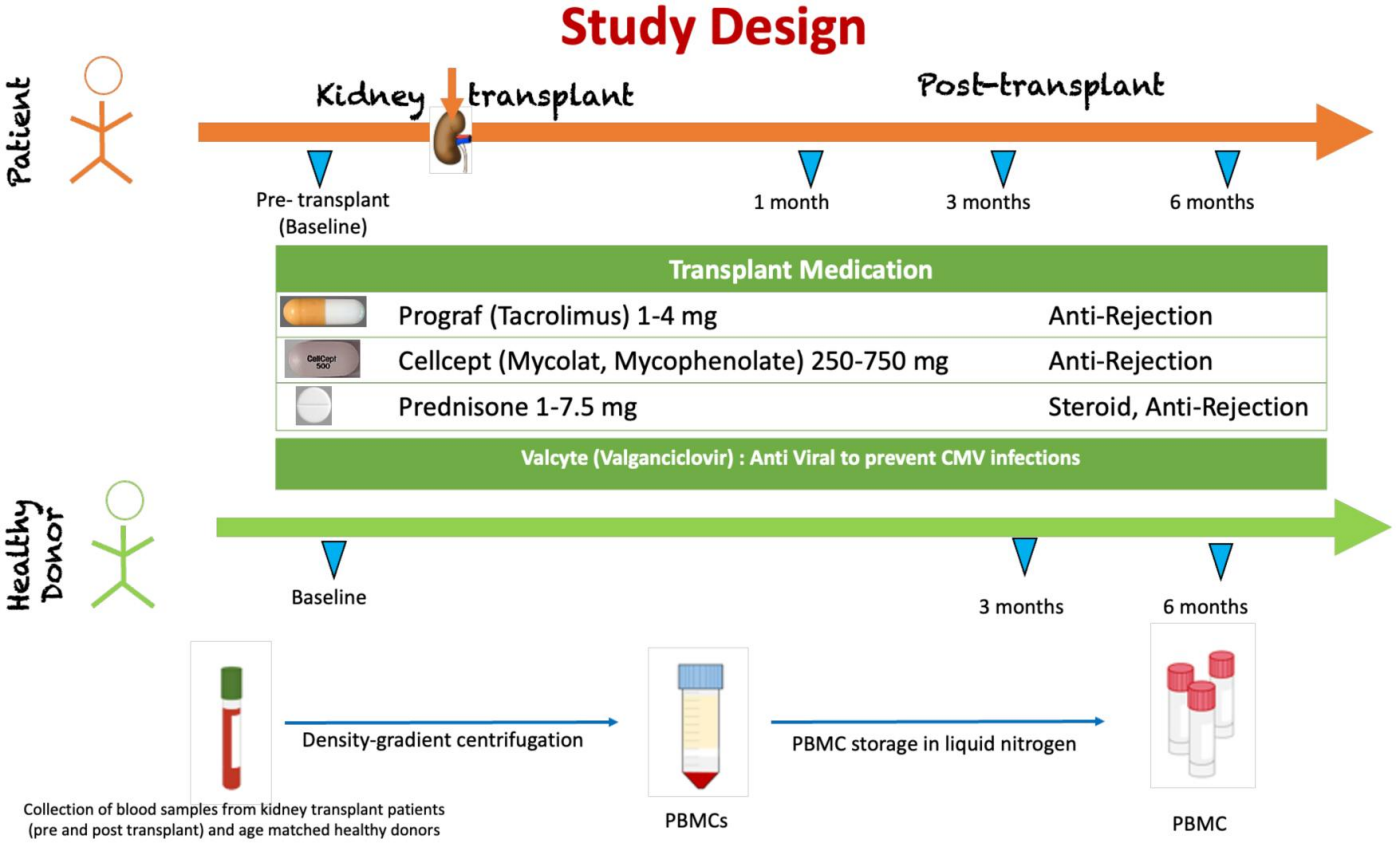
Study design. Samples were obtained from transplant patients and age-matched healthy donors at indicated time intervals. PBMCs were extracted using density-gradient centrifugation and stored in liquid nitrogen. Following transplantation, recipients were administered the medications listed in Table 1 for maintenance.

A total of 10 kidney transplant recipients (40% female) and 11 age-matched healthy control subjects (64% female) were recruited for the study under a protocol (IRB40027) approved by the Stanford Institutional Review Board. In this study, the 10 renal transplant patients received induction therapy of rabbit antithymocyte globulin (ATG) and/or methylprednisolone (Table 1). Transplant patients were started and maintained for 6 mo on standard doses of immunosuppressive drugs tacrolimus, mycophenolate mofetil and prednisone. Valganciclovir was administered prophylactically for cytomegalovirus. Blood samples from patients were obtained at baseline (prior to transplantation) and at 1, 3, and 6 mo post–renal transplantation. Blood samples from healthy control subjects were obtained at baseline, 3 mo, and 6 mo. Peripheral blood mononuclear cells (PBMCs) were isolated from blood of patients and healthy control subjects and stored in liquid nitrogen (Fig. 1). The demographic characteristics of the study population is summarized in Table 1. Detailed features and outcomes of the renal transplant recipients is outlined in Table S1.

**Table 1.** Demographics of study population

Demographic parameter	Patients	Healthy donors
Age, yr	31–71	31–59
Female	4 (40)	7 (64)
Total number of study subjects	10	11
Induction IS	Rabbit ATG, methylprednisolone	N/A
Viral prophylaxis	Valganciclovir (Valcyte)	N/A

Values are range, n (%), or n.

Abbreviations: IS, immunosuppression; N/A, not applicable.

### Extracellular and intracellular sample staining for CyTOF

**Figure 2.**
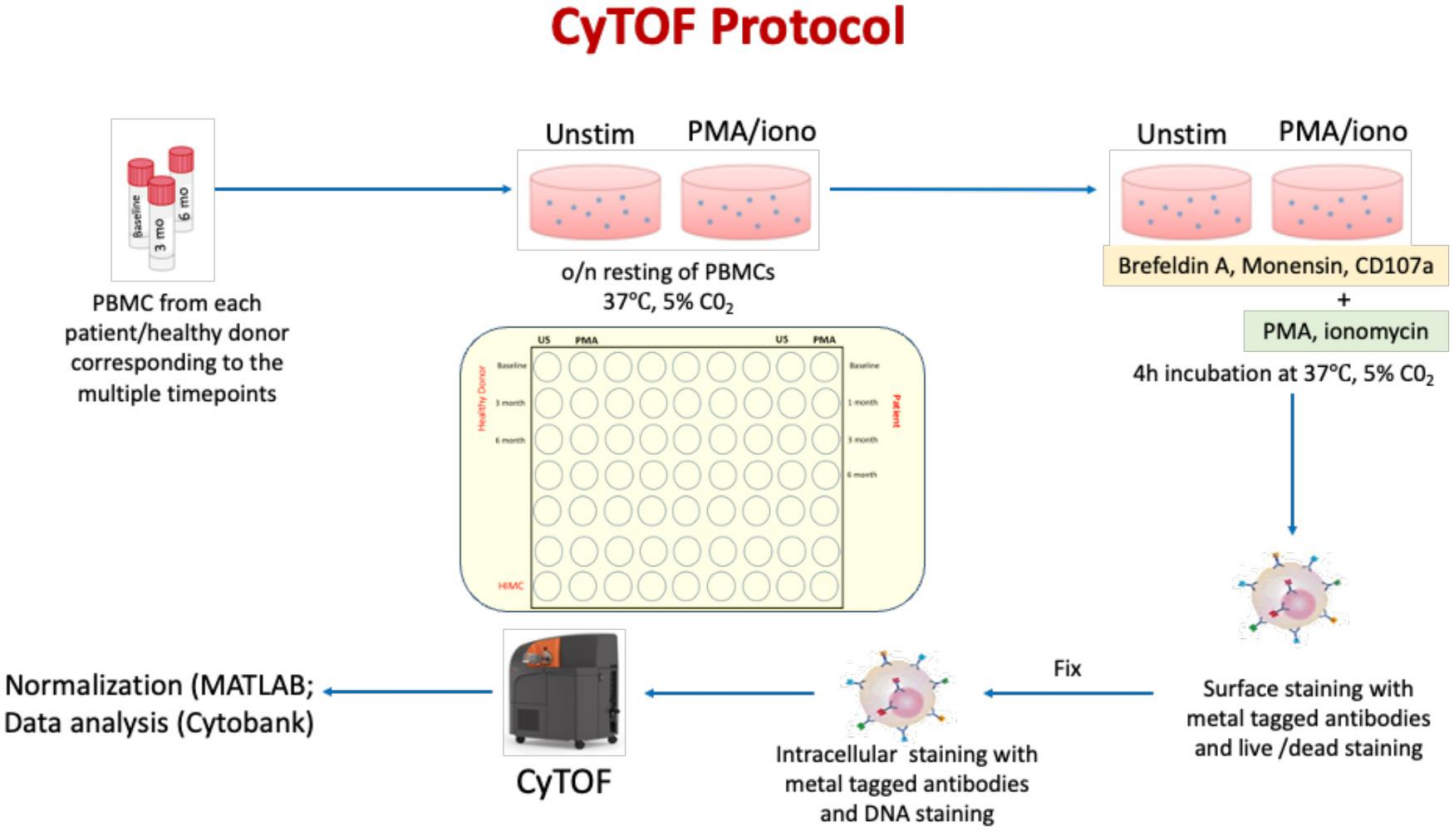
CyTOF protocol. A schematic illustrating the processing of PBMCs from both transplant patients and healthy donors for intracellular staining followed by the acquisition of samples on CyTOF. Subsequently, the acquired .fcs files underwent normalization using the MATLAB normalizer before being analyzed in Cytobank. o/n, overnight.

PBMCs from patients and healthy donors were processed for intracellular cytokine staining by CyTOF as previously described.^10^ Briefly, for each batch, frozen PBMCs corresponding to all the time points for a given patient and healthy donor were thawed in a 37 °C water bath, followed by 2 washes in warm complete medium (RPMI supplemented with penicillin-streptomycin and L-glutamine; HyClone [GE Healthcare Life Sciences]) containing benzonase nuclease (Novagen [Millipore]). PBMCs were then resuspended in complete medium (no benzonase nuclease), and cell viability and cell counts were obtained using the Vi-Cell XR cell viability analyzer (Beckman Coulter). After counting, samples from each time point were split into 2 experimental conditions: unstimulated and PMA (10 ng/mL) (Sigma-Aldrich) + ionomycin (1 μg/mL) (Sigma-Aldrich), with a maximum of 2 × 10^6^ cells per condition. Cells were plated and rested in 96-well U-bottom plates overnight at 37 °C, 5% CO_2_. After resting the cells, secretion inhibitors brefeldin A (5 μg/mL) and monensin (5 μg/mL) (Sigma-Aldrich) were added to both unstimulated and PMA + ionomycin conditions. Simultaneously, anti-CD107a antibody conjugated to 151Eu (Fluidigm) was also added to both conditions, at a concentration as recommended by the supplier. All samples were incubated for 4 h at 37 °C. Brefeldin A, monensin, PMA, and ionomycin were in CyPBS (10X phosphate-buffered saline [PBS] without heavy metal contaminants diluted 1:10 in MilliQ water; Rockland Immunochemicals). At the end of stimulation, a final concentration of 2 mM EDTA was added to each well and incubated for 15 min at room temperature. Cells were washed twice in CyFACS buffer (CyPBS supplemented with 2 mM EDTA, 0.1% bovine serum albumin, and 0.05% sodium azide). Cell surface antibody cocktail consisting of both preconjugated antibodies (Standard BioTools) and in-house conjugated antibodies (Table 2) was prepared in CyFACS and filtered using 0.1 μm spin filters (Millipore) to remove possible antibody aggregates before staining. A total of 70 μL of the antibody cocktail was then added to all the wells and cells were incubated for 45 min on ice. Cells were washed twice in CyFACS after surface staining, followed by live/dead staining (1:3,000 115In-DOTA Maleimide [5 mg/mL]) for 30 min on ice. Cells were washed 3 times with CyFACS and then fixed in 2% paraformaldehyde in PBS at 4 °C overnight. These fixed cells were permeabilized in 0.1% Triton X-100 for 15 min at room temperature (Sigma-Aldrich). An intracellular staining cocktail, also consisting of Standard BioTools preconjugated as well as in-house conjugated antibodies was prepared in 1X permeabilization buffer (eBioscience) and added to the cells for 45 min on ice (Table 2). After washing 3 times in CyFACS, cells were stained with Intercalator-Ir (Standard BioTools) diluted as per the manufacturer’s recommendations in 2% paraformaldehyde in PBS and incubated for 20 min at room temperature. Finally, cells were washed twice in CyFACS and 3 times in MilliQ water. EQ Four Element Calibration Beads from Standard BioTools were added as per the manufacturer’s directions prior to acquisition. Data were acquired on a Helios mass cytometer (Standard BioTools) in batches Fig. 2.

**Table 2.** CyTOF staining panel for phenotypic and functional analysis of immune cell subsets, with metal labels, antibody clone, and source.

Metal label	Specificity	Antibody clone	Source
**113Id**	CD57	HCD57 (BioLegend)	In-house
**115In**	live/dead		
**140Ce**	Beads		Fluidigm
**141Pr**	HLA-DR	G46-6, BD	In-house
**142Nd**	CD19	HIB19	Fluidigm
**143Nd**	IL-10	JES3-9D7 (BioLegend)	In-house
**144Nd**	1L-4	MPA-25D2	Fluidigm
**145Nd**	CD4	RPA-T4	Fluidigm
**146Nd**	CD8	RPA-T8	Fluidigm
**147Sm**	CD20	2H7	Fluidigm
**148Nd**	CD40	5C3 (BioLegend)	In-house
**149Sm**	CTLA-4	14D3 (eBioscience)	In-house
**150Nd**	MIP-1b	D21-351	Fluidigm
**151Eu**	CD107a	H4A3	Fluidigm
**152Sm**	TNFa	Mab11	Fluidigm
**153Eu**	CD45RA	HI100	Fluidigm
**154Sm**	CD3	UCHT1	Fluidigm
**155Gd**	CD28	L293 (BD)	In-house
**156Gd**	CD38	HB-7 (BD)	In-house
**157Gd**	CD25	MA251 (BD)	In-house
**158Gd**	CD33	WM53	Fluidigm
**159Tb**	GMCSF	BCD2-21C11	Fluidigm
**160Gd**	CD14	M5E2	Fluidigm
**161Dy**	IFNg	4S.B4 (eBioscience)	In-house
**162Dy**	CD69	MCA 1442	Fluidigm
**163Dy**	TCRgd	B1 (BioLegend)	In-house
**164Dy**	IL-17	N49-653	Fluidigm
**165Ho**	CD127	A019D5	Fluidigm
**166Er**	IL-2	MQ1-17H12	Fluidigm
**167Er**	CD27	L128	Fluidigm
**168Er**	CD40L	24-31	Fluidigm
**169Tm**	CCR7	150503 (R&D Systems)	In-house
**170Er**	PD1	EH12.1, BD	In-house
**171Yb**	Granzyme B	GB11	Fluidigm
**172Yb**	pH2A.X	JBW301 (Millipore)	In-house
**173Yb**	Perforin	B-D48 (Abcam)	In-house
**174Yb**	CD21	Bu32 (BioLegend)	In-house
**175Lu**	PD-L1	29E.2A3	Fluidigm
**176Yb**	CD56	NCAM16.2	Fluidigm
**193Ir**	DNA Intercalator		Fluidigm
**195Ir**	DNA Intercalator		Fluidigm
**209Bi**	CD16	3G8	Fluidigm

### CyTOF data preprocessing and analysis

Data were obtained in the form of .fcs files from the Helios instrument and normalized using the Nolan Lab MATLAB normalizer freely available on Github (https://github.com/nolanlab/bead-normalization/releases). The MATLAB normalizer uses the EQ calibration bead channel signal intensities to normalize signal intensities from the channels being analyzed. Normalized .fcs files were then uploaded to Cytobank (www.cytobank.org) for sequential gating Fig. 2. The iridium isotopes from the DNA intercalator as well as 140Ce beads and event length parameters were used to discern intact singlets from debris and cell aggregates. Live intact singlets were then identified using the 115In-DOTA Maleimide live/dead staining. From the live intact singlets we gated distinct cell lineage subsets such as CD4^+^ T cells (CD3^+^CD4^+^), CD8^+^ T cells (CD3^+^CD8^+^), double negative (DN) T cells (CD3^+^CD4^−^CD8^−^), γδ T cells (CD3^+^CD4^−^CD8^−^TCRγδ^+^), natural killer T (NKT) cells (CD3^+^CD56^+^), T regulatory cells (Tregs) (CD3^+^CD4^+^CD127^lo^CD25^+^), B cells (CD3^−^CD19^+^CD20^+^), natural killer (NK) cells (CD3^−^CD56^+^CD16^+/low^), monocytes (CD33^+^CD14^+^),^11^ and plasmablasts (CD19^+^CD27^high^CD38^high^)^12^ by supervised gating in Cytobank (Fig. S1). Data shown are in the form of relative percentages of the parent cell population as derived from Cytobank gating. GraphPad Prism 8.0 (GraphPad Software) was used to plot graphs.

### Statistical analysis

One-way analysis of variance (ANOVA) (Kruskal-Wallis test) statistical analysis within GraphPad Prism 9.5.0 (GraphPad Software) was used to compare healthy donors and patients at each visit. Multiple comparison corrections were performed using Dunn’s test to generate adjusted *P* values (*P < *0.05, *P < *0.01, *P < *0.001). The error bars represent the mean ± SEM. For the memory T cell subsets, we performed 2-way ANOVA (or mixed model). To determine statistical differences in the distribution of the memory T subsets, we performed Dunnett’s test for multiple comparisons to generate adjusted *P* values (*P < *0.05, *P < *0.01, *P < *0.001). Data are represented as mean ± SEM.

Changes due to immunosuppressive therapy were compared between baseline and post-transplantation time points within the patient cohort. To determine phenotypic and functional changes, 1-way ANOVA (nonparametric or mixed model) was performed using Dunnett’s multiple comparisons test between 1, 3, and 6 mo post-transplantation vs baseline. To determine differences in the distribution of the memory T cell subsets, we applied 2-way ANOVA (or mixed model) statistical analysis within the GraphPad Prism 9.5.0. Multiple comparisons between 1, 3, and 6 mo post-transplantation vs baseline were performed using Dunnett’s test to generate adjusted *P* values (*P < *0.05, *P < *0.01). Data are represented as mean ±SEM.

## Results

We carried out a comprehensive immune profiling study to determine differences in immune signatures pre– and post–renal transplantation in patients as compared with age-matched healthy donors. We used mass cytometry (CyTOF) to profile the PBMCs in renal allograft recipients to gain a deeper understanding of their phenotypic and functional immune landscape.

To compare the immune profiles of the 10 transplant recipients with what would be expected to be healthy profiles, PBMCs from 11 age-matched healthy donors were procured at baseline, 3 mo, and 6 mo. An average of the data points at each time point for the healthy donors was calculated for analysis, comparing changes between healthy donors and transplant patients at baseline and at each time point of the transplant patients. For the transplant patients, baseline samples were obtained prior to transplantation during primary kidney disease and prior to receiving immunosuppression.

### Immunosuppressive treatment induces changes in CD56^dim^CD16^+^ NK cells and plasmablasts in renal transplant patients compared with the healthy donors

**Figure 3.**
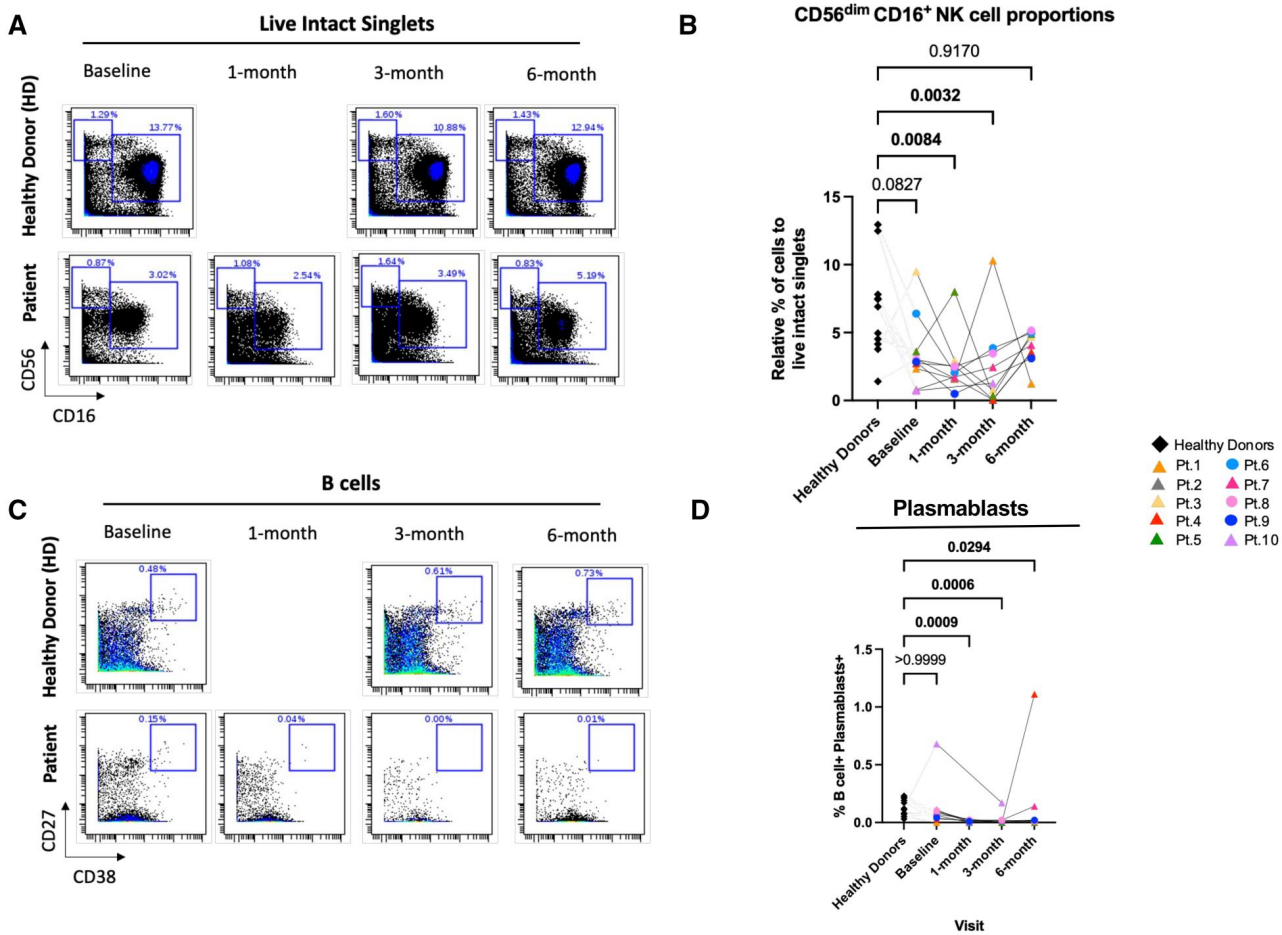
Phenotypic changes were observed in patients after transplantation compared with the healthy donors. CyTOF analysis by 1-way ANOVA showed significant decrease in the cell frequencies of unstimulated CD56^dim^CD16^+^ NK cells of transplant patients 1 and 3 mo post-transplantation compared with the healthy donors. (A) Representative dot plot showing gating schematic of CD56^dim^CD16^+^ NK cells from live intact singlets. (B) Frequencies of CD56^dim^CD16^+^ NK cells normalized to live intact singlets. Cell frequencies of plasmablasts were also significantly decreased post-transplantation compared with the healthy donors. (C) Representative dot plot showing gating schematic of plasmablasts from B cells and (D) frequencies of plasmablasts normalized to B cells. For patients, baseline, n = 10; 1 mo, n = 9; 3 mo, n = 8; and 6 mo, n = 9. For healthy control subjects, n = 11 (average of baseline, 3 mo, and 6 mo). Graphs show mean ± SEM for each subset. Adjusted *P* values were calculated by 1-way ANOVA using Dunn’s multiple comparisons test. Cohort representation: diamond indicates healthy donors; triangle indicates patients who received rabbit ATG induction therapy; circle indicates patients who did not receive rabbit ATG induction therapy. Pt, patient.

To identify phenotypic differences between the renal transplant recipients and corresponding healthy age-matched donors, we assessed the frequencies of each cell population between the transplant recipients across all time points and healthy donors (average of all time points). Figure 3A is a representative dot plot showing the gating strategy for the CD56^dim^CD16^+^ NK cells from the live intact cells. One-way ANOVA analysis showed the cell frequencies of CD56^dim^CD16^+^ NK cells were significantly reduced in the transplant recipients at 1 and 3 mo after transplantation compared with age-matched healthy donors (*P < *0.01) (Fig. 3B). At 6 mo post-transplantation, there was a partial recovery in the cell frequencies of CD56^dim^CD16^+^ NK cells, such that the difference from control subjects was no longer statistically significant.

Our analysis also showed a significant reduction in plasmablasts in the renal recipients at all time points (1, 3, and 6 mo) after transplantation. Plasmablasts were gated from B cells, as shown in the representative dot plot (Fig. 3C). Figure 3D shows significant reduction at all time points after transplantation in the graft recipients compared with the healthy donors (*P < *0.05, *P < *0.001). Interestingly, at 6 mo, plasmablasts frequencies in patient 4 (Fig. 3D, red triangle) and patient 7 (Fig. 3D, pink triangle) were elevated. Patient 4 rejected the graft at 6 mo post-transplantation and patient 7 displayed T cell mediated rejection at 6 mo and was treated with a pulse of oral steroids for 3 d (Table S1). For the other major immune subsets, including T cells, CD4^+^ and CD8^+^ T cells, γδ T cells, NKT cells, Tregs, B cells, CD56^bright^ NK cells, and monocytes, there were no significant differences in the cell frequencies between the transplant recipients and healthy donors at any time point (Fig. S2A–I).

### Changes in CD8 memory cell subsets in renal transplant recipients compared with age-matched healthy donors

**Figure 4.**
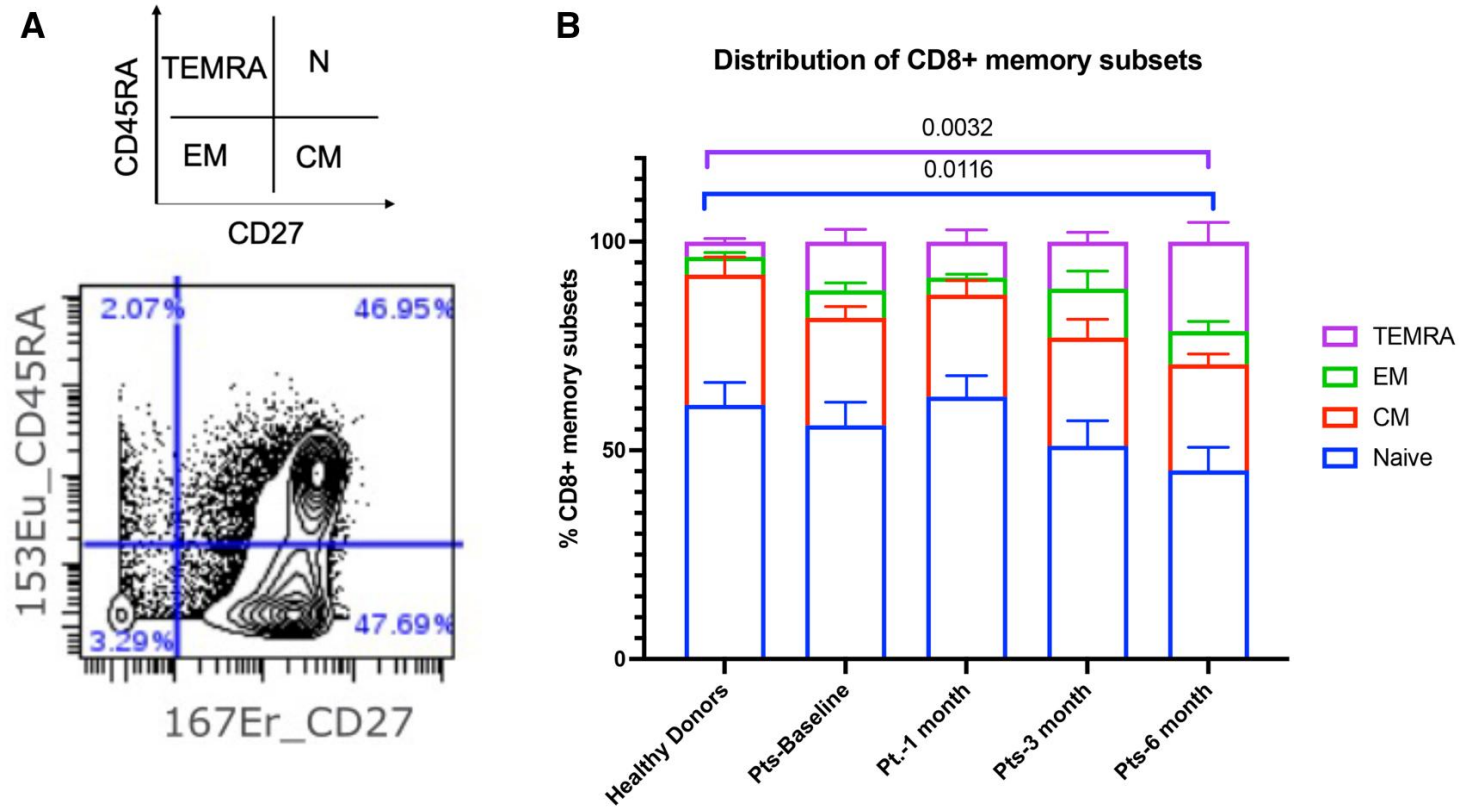
Renal transplant patients have lower frequencies of CD8^+^ naïve memory T cells and higher frequencies of CD8^+^ TEMRA memory T cells 6 mo post-transplantation. CyTOF data showing classification of CD8^+^ memory T subsets using CD45RA and CD27 expression—CD27^+^CD45RA^+^ cells are naïve (N) CD8^+^ T cells, CD27^−^CD45RA^+^ cells are TEMRA cells, CD27^−^CD45RA^−^ cells are EM cells, and CD27^+^CD45RA^−^ cells are CM cells. (A) Differential distribution of CD8^+^ T cell memory subsets between healthy donors and patients at baseline, 1 mo, 3 mo, and 6 mo post-transplantation (B). For patients, baseline, n = 10; 1 mo, n = 9; 3 mo, n = 8; and 6 mo, n = 9. For healthy control subjects, n = 11 (average of baseline, 3 mo, and 6 mo). Graphs show mean ± SEM for each subset. Adjusted *P* values were calculated using Dunnett’s multiple comparisons test. Pt, patient.

Because T cells play a central role in graft acceptance or rejection, we next examined the phenotypic profile of the naïve and memory T cell subsets across all time points in the transplant patients vs the healthy donors. To identify the memory T cell subsets, expression of CD45RA in conjunction with CD27 was used. Figure 4A shows the differential distribution of naïve (CD45RA^+^CD27^+^), central memory (CM) (CD45RA^−^CD27^+^), effector memory (EM) (CD45RA^−^CD27^−^), and terminally differentiated effector memory (TEMRA) (CD45RA^+^CD27^−^) cells. At 6 mo post-transplantation, the expression of CD8^+^ naïve T cells was significantly reduced and CD8^+^ TEMRA T subset was significantly increased in the transplanted patients compared with the healthy donors (*P < *0.05 and *P < *0.01, respectively) (Fig. 4B). No significant differences in the naïve and TEMRA subsets were observed at the other time points between the renal transplant recipients and healthy donors (Fig. 4B). CD8^+^ CM T cells and EM T cells across all time points tended to be lower in the transplant recipients compared with the healthy control subjects, although not statistically significantly so (Fig. 4B). No significant differences were observed in the differential distribution of the CD4^+^ T cell memory subsets between the transplant patients and healthy donors across all time points measured (data not shown).

### Differential cytokine production by γδ T cells, CD8^+^ T cells, and Tregs post-transplantation in renal recipients

**Figure 5.**
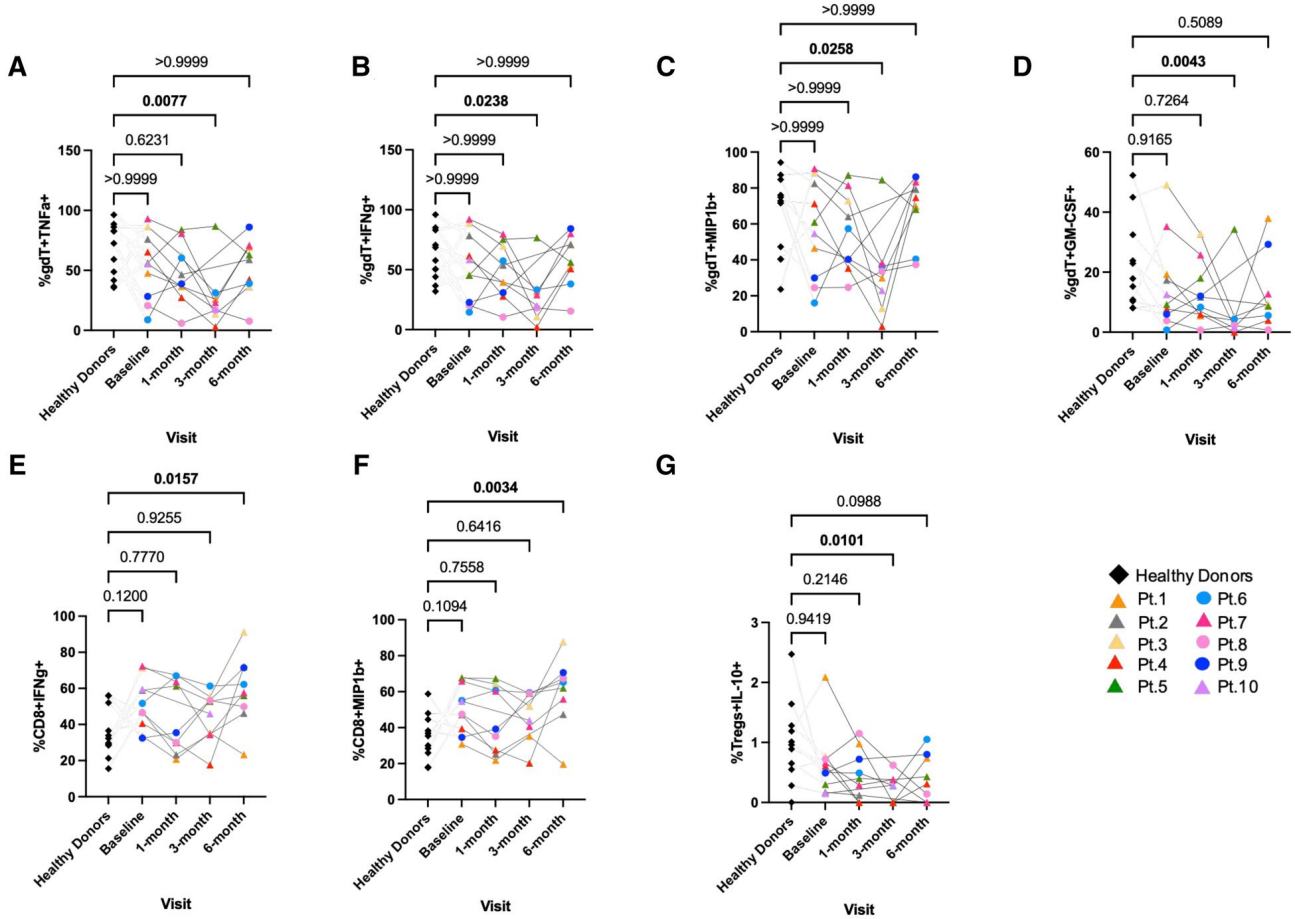
Differential cytokine production by γδ T cells, CD8^+^ T cells, and Tregs post-transplantation following stimulation with PMA + ionomycin ex vivo. Significantly changed cell frequencies of (A) TNFα^+^ (γδ T cells), (B) IFNγ^+^ (γδ T cells), (C) MIP-1β^+^ (γδ T cells), (D) GM-CSF^+^ (γδ T cells), (E) IFNγ^+^ (CD8^+^ T cells), (F) MIP-1β^+^ (CD8^+^ T cells), and (G) IL-10^+^ (Tregs) are shown. Data are represented as a percentage of the parent population. For patients, baseline, n = 10; 1 mo, n = 9; 3 mo, n = 8; and 6 mo, n = 9. For healthy control subjects, n = 11 (average of baseline, 3 mo, and 6 mo). Graphs show mean ± SEM for each subset. Adjusted *P* values were calculated using 1-way ANOVA and Dunn’s multiple comparisons test. Cohort representation: diamond indicates healthy donors; triangle indicates patients who received rabbit ATG induction therapy; circle indicates patients who did not receive rabbit ATG induction therapy. Pt, patient.

To further document immune alterations in transplant recipients, we examined the expression of various functional markers at baseline, 1 mo, 3 mo, and 6 mo after transplantation between patients and in healthy donors. Three months following renal transplantation, production of tumor necrosis factor α (TNFα) (*P < *0.01) (Fig. 5A), interferon γ (IFNγ) (*P < *0.05) (Fig. 5B), MIP-1β (*P < *0.05) (Fig. 5C), and granulocyte-macrophage colony-stimulating factor (GM-CSF) (*P < *0.01) (Fig. 5D) by γδ T cells was significantly reduced compared with healthy control subjects. In the CD8^+^ T cells, IFNγ and MIP-1β expression was significantly higher in patients 6 mo after transplantation (*P < *0.05 and *P < *0.01, respectively) (Fig. 5E, F), and interleukin (IL)-10 expression by Tregs was significantly reduced 3 mo after transplantation compared with control subjects (*P < *0.05) (Fig. 5G).

### CD8^+^ T cells memory subsets show differential cytokine expression in patients following renal transplantation

**Figure 6.**
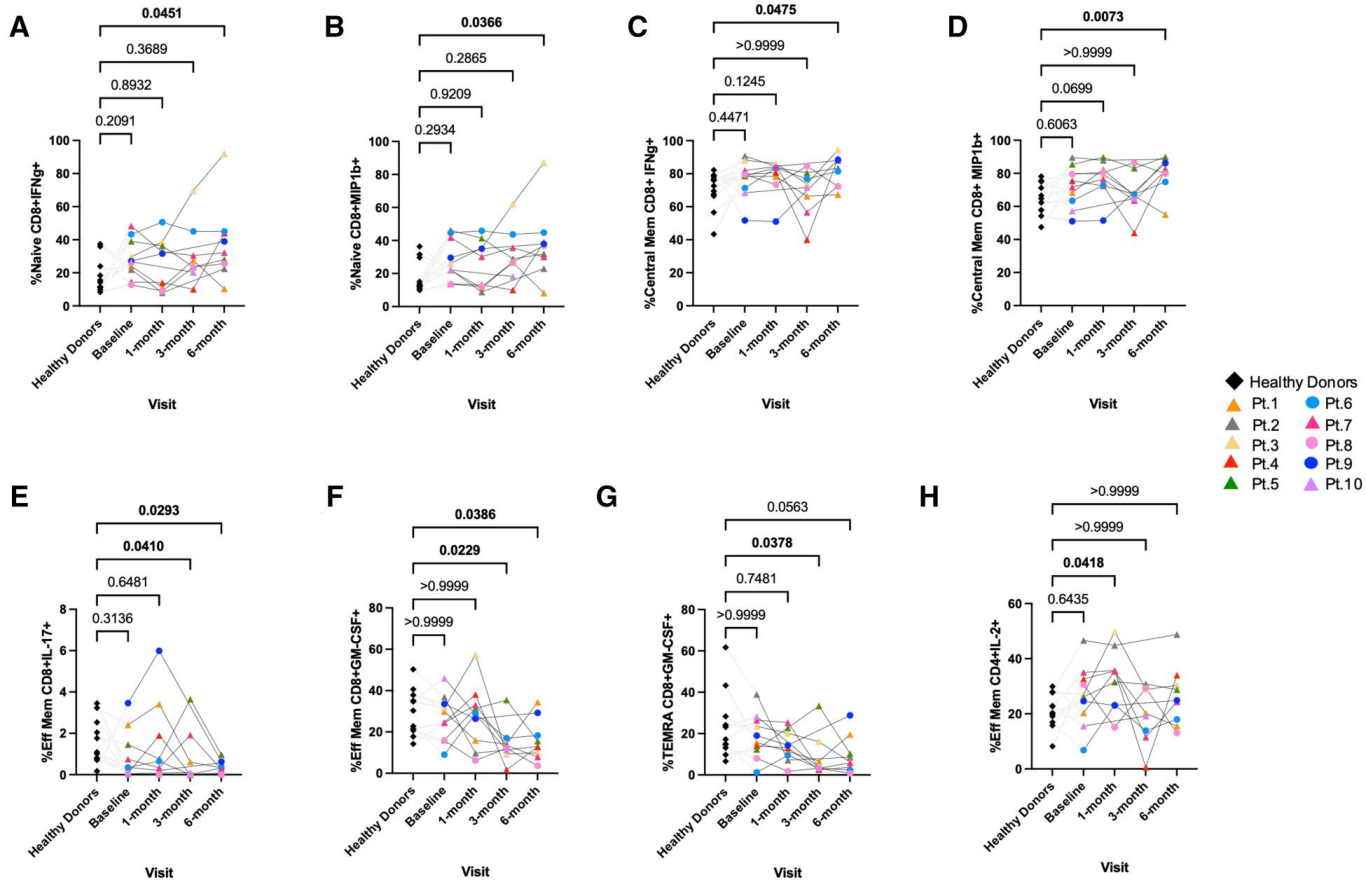
Cytokine expression in CD8^+^ memory T cells after PMA + ionomycin stimulation ex vivo. One-way ANOVA analysis showed (A) IFNγ (CD8^+^ naïve T cells), (B) MIP-1β (CD8^+^ naïve T cells), (C) IFNγ (CD8^+^ CM T cells), and (D) MIP-1β (CD8^+^ CM T cells) expression to be significantly increased in renal transplant recipients. Expression of (E) IL-17 (CD8^+^ EM T cells), (F) GM-CSF (CD8^+^ EM T cells), and (G) GM-CSF (CD8^+^ TEMRA T cells) was significantly decreased in renal transplant recipients. (H) Expression of IL-2 (CD4^+^ EM T cells) was significantly increased in renal transplant patients. Data are represented as a percentage of the parent population. For patients, baseline, n = 10; 1 mo, n = 9; 3 mo, n = 8; and 6 mo, n = 9. For healthy control subjects, n = 11 (average of baseline, 3 mo, and 6 mo). Graphs show mean ± SEM for each subset. Adjusted *P* values were calculated using Dunn’s multiple comparisons test. Cohort representation: diamond indicates healthy donors; triangle indicates patients who received rabbit ATG induction therapy; circle indicates patients who did not receive rabbit ATG induction therapy. Pt, patient.

Functional changes depend on the stage of T cell maturity. As we observed phenotypic differences in the CD8^+^ memory T subsets as well as functional changes for total CD8^+^ T cells, we also examined if there were functional differences within each CD8^+^ memory T cell subset between patients and healthy donors. We observed a statistically significant increase of IFNγ^+^ and MIP-1β^+^ expression in CD8^+^ naïve and CM T cells in patients at 6 mo post–renal transplantation relative to control subjects (*P < *0.05 and *P < *0.01, respectively) (Fig. 6A–D), as were also observed in total CD8^+^ T cells (Fig. 5E, F). Additionally, we observed a significant decrease in the expression of IL-17 on CD8^+^ EM T cells in the renal transplant recipients 3 and 6 mo after transplantation (*P < *0.05) (Fig. 6E). GM-CSF expression was also significantly reduced in CD8^+^ EM T cells at 3 and 6 mo post-transplantation (*P < *0.05) (Fig. 6F) and in the CD8^+^ TEMRA T cells 3 mo after transplantation in renal recipients (*P < *0.05) (Fig. 6G). In the CD4^+^ memory T subsets, the expression of IL-2 on CD4^+^ EM T cells was significantly increased 1 mo after transplantation (*P < *0.05) (Fig. 6H).

### Decreased expression of costimulatory molecules by CD8^+^ T cells and γδ T cells post-transplantation in renal transplant recipients

**Figure 7.**
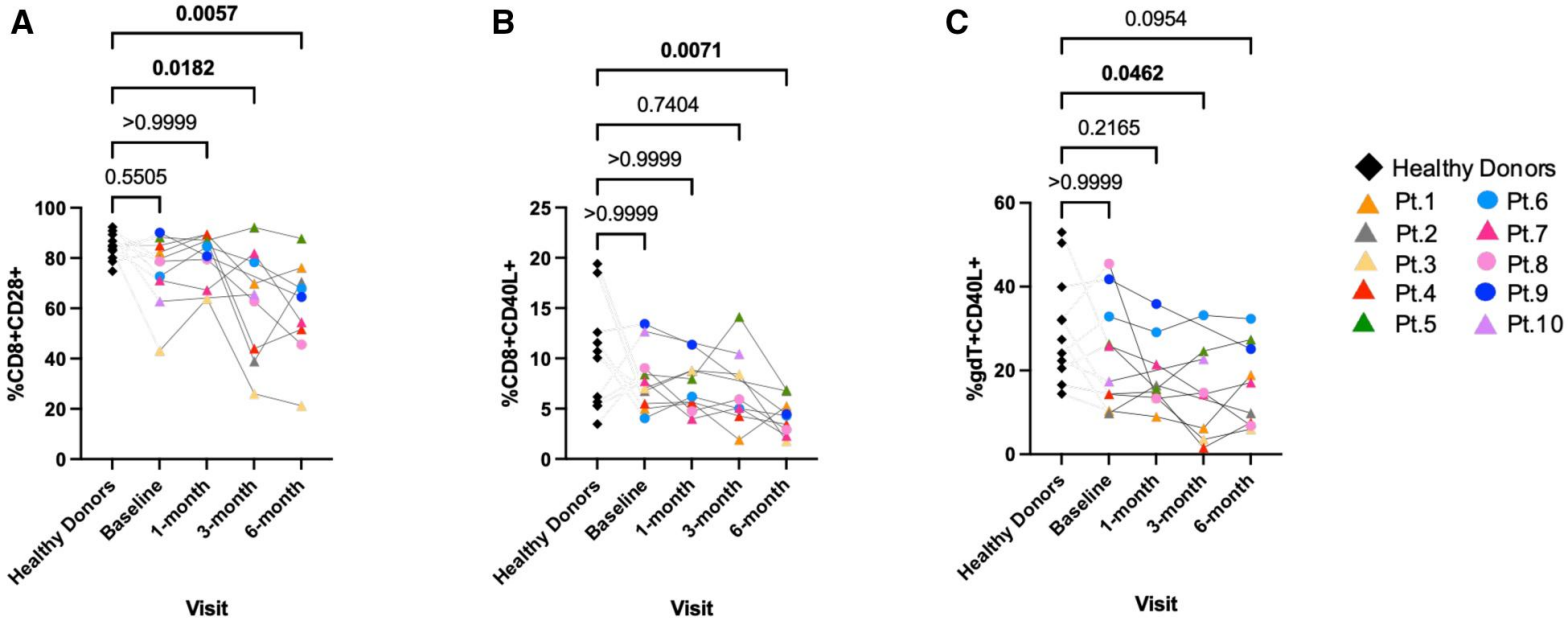
Decreased expression of costimulatory molecules in renal transplant recipients. One-way ANOVA showed significant decreased cell frequencies of (A) CD28 (unstimulated) and (B) CD40L (PMA + ionomycin stimulated) on CD8+ T cells and (C) CD40L (PMA + ionomycin stimulated) on γδ T cells in transplant recipients compared with healthy donors. Data are represented as a percentage of the parent population. For patients, baseline, n = 10; 1 mo, n = 9; 3 mo, n = 8; and 6 mo, n = 9. For healthy control subjects, n = 11 (average of baseline, 3 mo, and 6 mo). Graphs show mean ± SEM for each subset. Adjusted *P* values were calculated using Dunn’s multiple comparisons test. Cohort representation: diamond indicates healthy donors; triangle indicates patients who received rabbit ATG induction therapy; circle indicates patients who did not receive rabbit ATG induction therapy. Pt, patient.

Unstimulated PBMCs showed frequencies of CD8^+^ CD28^+^ T cells to be significantly lower at 3 and 6 mo after transplantation in the renal allograft recipients (*P < *0.05 and *P < *0.01, respectively) (Fig. 7A). Following PMA + ionomycin stimulation, CD40 ligand (CD40L) expression in CD8^+^ T cells and γδ T cells was also found to be significantly less at 6 mo and 3 mo, respectively, post-transplantation (*P < *0.01 and *P < *0.05, respectively) (Fig. 7B, C) in the transplant recipients relative to healthy control subjects.

### Increased cytotoxic activity from CD8^+^ and CD56^bright^ NK cells in renal transplant recipients

**Figure 8.**
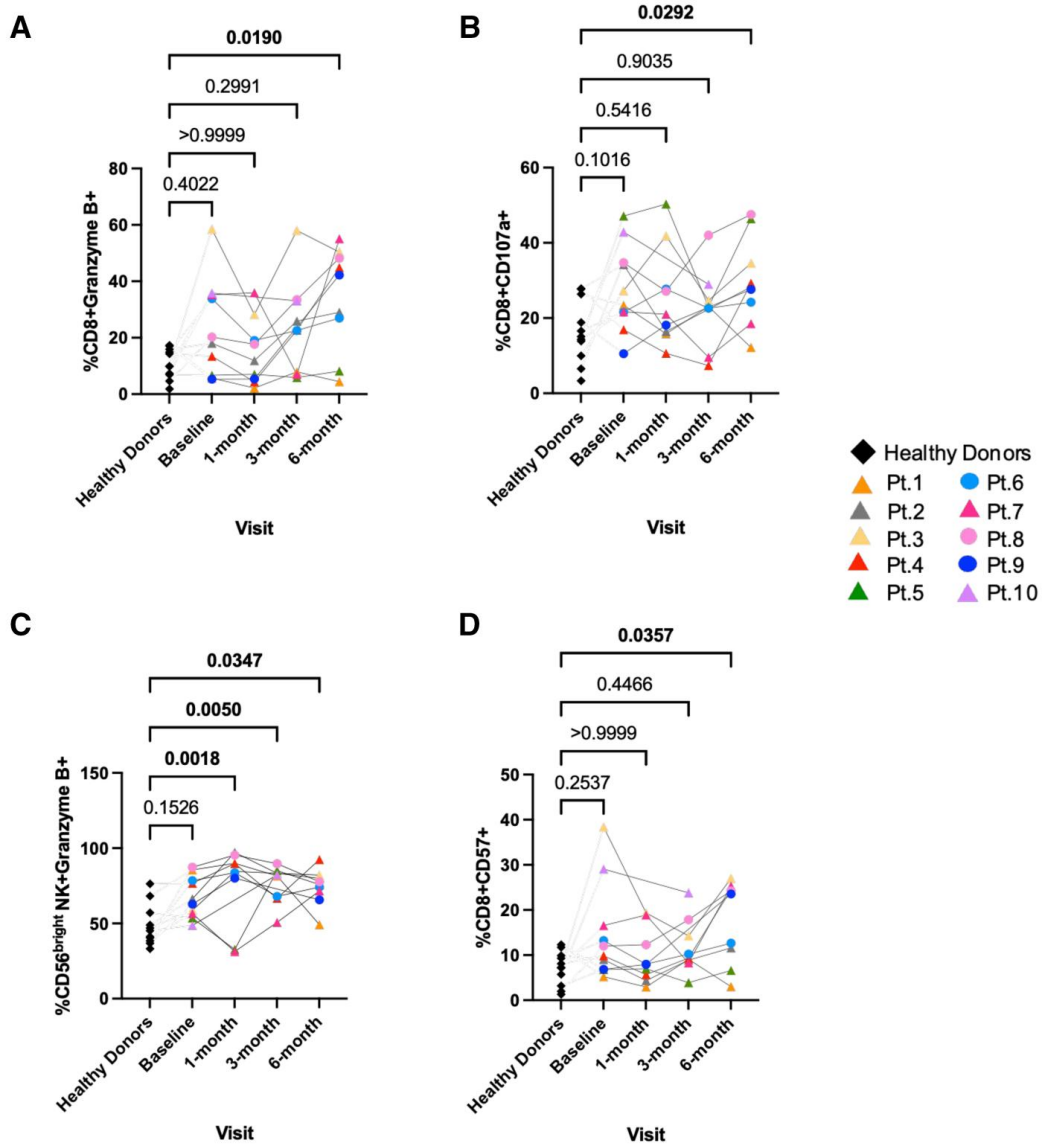
Granzyme B, CD107a, and CD57 expression in renal transplant recipients. One-way ANOVA showed significant increased cell frequencies of granzyme B on (A) unstimulated CD8^+^ T cells and (C) CD56^bright^ NK cells, (B) CD017a on PMA + ionomycin stimulated CD8^+^ T cells, and (D) CD57 on unstimulated CD8^+^ T cells in transplant recipients compared with healthy donors. Data are represented as a percentage of the parent population. For patients, baseline, n = 10; 1 mo, n = 9; 3 mo, n = 8; and 6 mo, n = 9. For healthy control subjects, n = 11 (average of baseline, 3 mo, and 6 mo). Graphs show mean ± SEM for each subset. Adjusted *P* values were calculated using Dunn’s multiple comparisons test. Cohort representation: diamond indicates healthy donors; triangle indicates patients who received rabbit ATG induction therapy; circle indicates patients who did not receive rabbit ATG induction therapy. Pt, patient.

Granule exocytosis is one of the most potent cell mediated death mechanisms used by CD8^+^ T cells and NK cells.^13^ In the renal transplantation setting, evaluating the expression of lytic granules in PBMCs or graft tissue can aid in assessing the efficacy of early graft acceptance or rejection.^14^ To examine the cytotoxic function of PBMCS from renal transplant recipients, we evaluated the expression of lytic granules perforin and granzyme B. We did not observe any significant changes in the expression of perforin by CD8^+^ T cells and NK cells (data not shown). However, our data show that unstimulated CD8^+^ T cells of patients have increased expression of granzyme B 6 mo after transplantation (*P < *0.05) (Fig. 8A). This was coupled with an increase in CD107a expression in the stimulated CD8^+^ T cells (*P < *0.05) (Fig. 8B). Of interest, unstimulated CD56^bright^ NK cells of the transplant recipients showed significant increases in granzyme B expression at all time points post-transplantation (*P < *0.05 and *P < *0.01) (Fig. 8C).

CD57 is an established biomarker of mature CD8^+^ T cells and NK cells and is used to identify differentiated T cells with reduced proliferative capacity. The expression of CD57 on lymphocytes correlates with their inability to proliferate and their high cytotoxic potential.^15^ Studies reported have also shown monoclonal expansion of CD8^+^ CD57^+^ T lymphocytes in renal transplant patients with no allograft rejection or viral infections.^16^ The findings from our CyTOF analysis on unstimulated PBMCs showed a significant increase in CD57 expression on CD8^+^ T cells at 6 mo post-transplantation (*P < *0.05) (Fig. 8D) in the renal transplant recipients compared with the healthy donors. We also observed a statistically significant decrease in CD57 expression on NKT cells at 3 mo post-transplantation (*P < *0.01) (Fig. S3).

### Phenotypic differences post-transplantation vs baseline in renal transplant recipients

**Figure 9.**
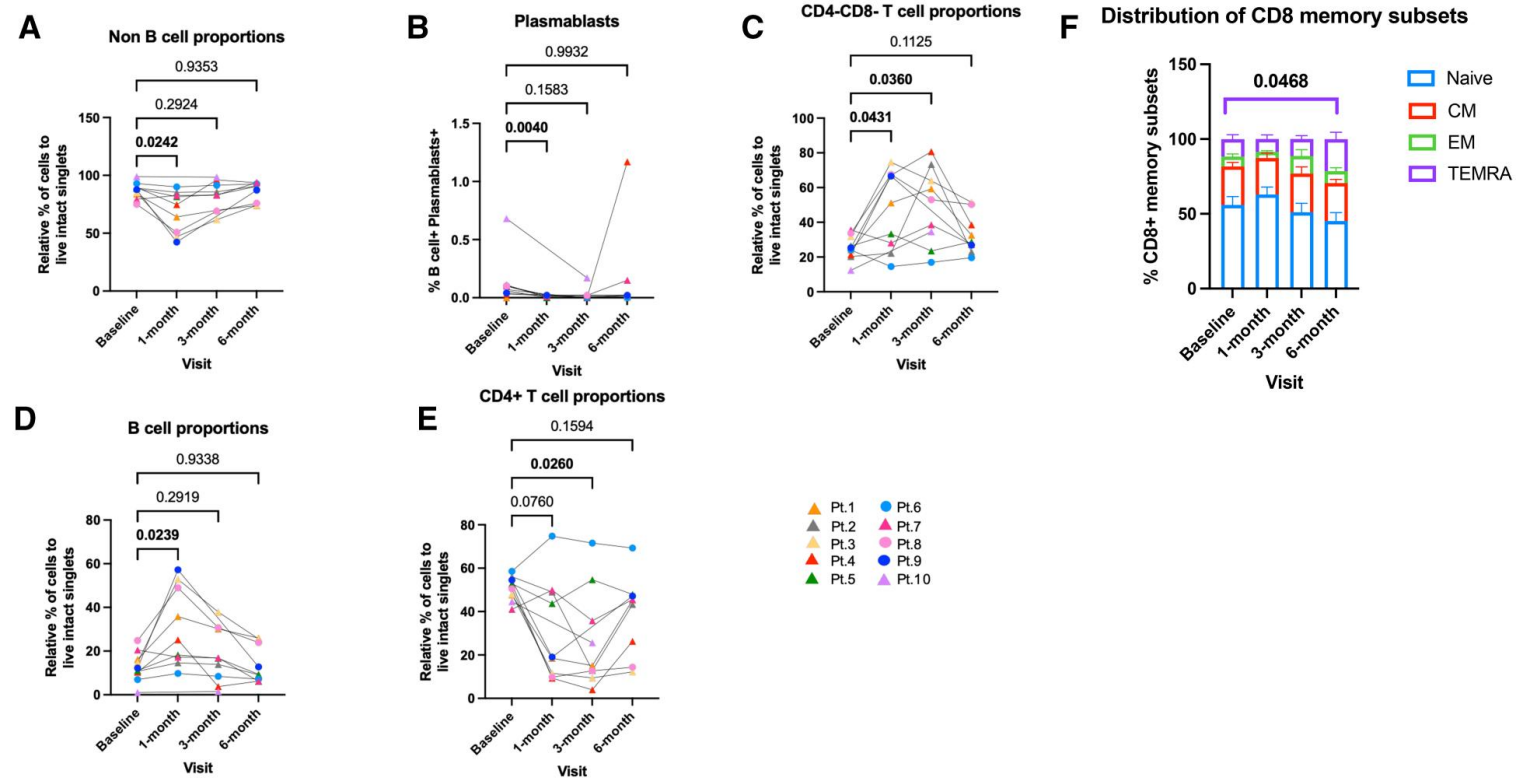
Phenotypic differences post-transplantation vs baseline in renal transplant recipients. CyTOF analysis by 1-way ANOVA using Dunnett’s multiple comparisons test showed a significant decrease in the cell frequencies of (A) unstimulated non–B cells normalized to live intact singlets. (B) Plasmablasts from B cells in renal transplant patients 1 mo post-transplantation compared with their baseline. (C) Cell frequencies of CD4^−^CD8^−^ T cells. (D) B cells normalized to live intact singlets significantly increased, while (E) cell frequencies of CD4^+^ T cells were significantly decreased in renal transplant patients post-transplantation compared with their baseline. Classification of CD8^+^ memory T subsets using CD45RA and CD27 expression was performed applying 2-way ANOVA using Dunnett’s multiple comparisons test. CD27^+^CD45RA^+^ cells are naïve CD8^+^ T cells (N), CD27^−^CD45RA^+^ cells are TEMRA cells, CD27^−^CD45RA^−^ cells are EM cells, and CD27^+^CD45RA^−^ cells are CM cells. (F) Differential distribution of CD8^+^ T cell memory subsets at baseline, 1 mo, 3 mo, and 6 mo post-transplantation showed a significant increase in the TEMRA subset at 6 mo post-transplantation vs baseline. For patients: baseline, n = 10; 1 mo, n = 9; 3 mo, n = 8; and 6 mo, n = 9. Graphs show mean ± SEM. Cohort representation: triangle indicates patients who received rabbit ATG induction therapy; circle indicates patients who did not receive rabbit ATG induction therapy. Pt, patient.

We were also interested in identifying changes within the renal transplant cohort over time by analyzing effects of the immunosuppressive therapy at all visits (i.e. 1, 3, and 6 mo post-transplantation vs baseline). Our analysis showed that at 1 mo post-transplantation there was a significant reduction in the frequency of non–B cells (CD3^−^CD19^−^CD20^−^) (*P < *0.05) and plasmablasts (*P < *0.01) (Fig. 9A, B), which could be due to induction with ATG.^17–19^ Cell frequencies of the CD4^−^CD8^−^ T cell and B cell proportions were significantly higher 1 mo post-transplantation compared with baseline (*P < *0.05). While CD4^−^CD8^−^ T cells sustained a significant increase in cell frequencies at 3 mo post-transplantation vs baseline (*P < *0.05), CD4^+^ T cells showed a significant decrease 3 mo post-transplantation (*P < *0.05) (Fig. 9C, E). We did not observe any statistically significant changes post-transplantation vs baseline for the other major immune subsets such as CD8^+^ T cells, γδ T cells, NKT cells, Tregs, CD56^dim^CD16^+^ NK and CD56^bright^ NK cells, or monocytes in the renal transplant recipients (data not shown). Next, we analyzed the memory T subsets to determine differences in the phenotypic memory subset profile post-transplantation vs baseline. The expression of CD45RA and CD27 markers was used to define the memory subsets as described in Fig. 2. Six months post-transplantation, the CD8^+^TEMRA subset (CD45RA^+^CD27^−^) was significantly increased compared with baseline (*P < *0.05) (Fig. 9F). No statistically significant differences were found for CD8^+^ naïve cells or the other CD8^+^ memory subsets (CM, EM), or the CD4^+^ memory T subsets (data not shown).

### Decreased cytokine profile post-transplantation vs baseline

**Figure 10.**
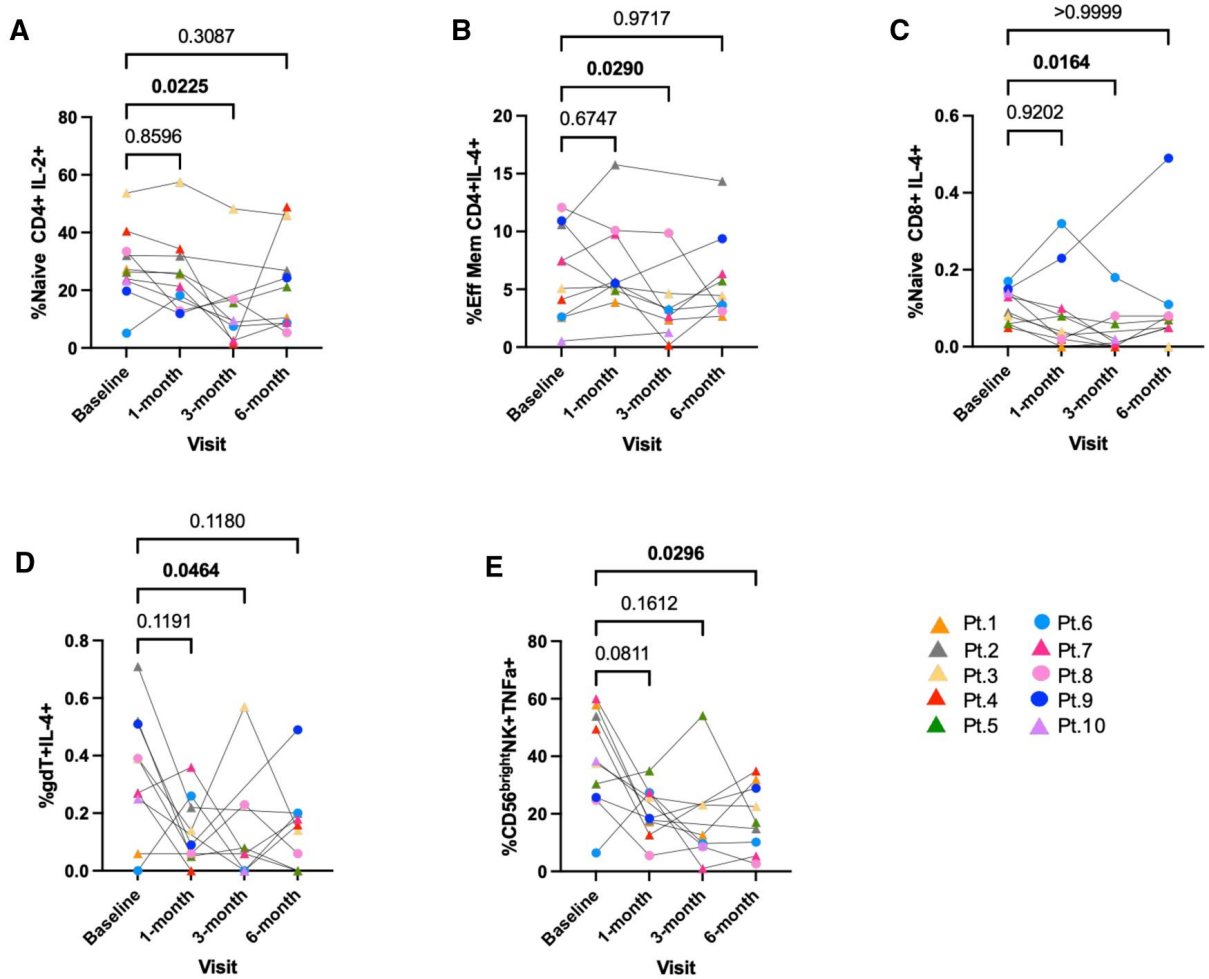
Decreased cytokine profile post-transplantation vs baseline following 4 h stimulation with PMA + ionomycin ex vivo. Frequencies of (A) IL-2+ (naïve CD4^+^ T cells), (B) IL-4+ (EM, CD4^+^ T cells), (C) IL-4^+^ (naïve CD8^+^ T cells), (D) IL-4^+^ (γδ T cells), and (E) TNFα^+^ (CD56^bright^ NK cells) are shown. Data are represented as a percentage of the parent population. For patients, baseline, n = 10; 1, 3, and 6 mo, n = 9. Graphs show mean ± SEM for each cell population. Adjusted *P* values were calculated by 1-way ANOVA (and nonparametric or mixed) using Dunnett’s multiple comparisons test. Cohort representation: triangle indicates patients who received rabbit ATG induction therapy; circle indicates patients who did not receive rabbit ATG induction therapy. Pt, patient.

To examine functional changes post-transplantation, we first assessed the cytokine profile (after in vitro stimulation with PMA + ionomycin for 4 h) for a total of 8 intracellular cytokines. Poststimulation, we did not find any significant changes in the cytokine profile 1 mo after transplantation. Three months post-transplantation, significant decreases in the expression of CD4^+^ naïve IL-2^+^ cells and IL-4^+^ expression in the CD4^+^ EM, CD8^+^ naïve, and γδ T cells were observed (*P < *0.05) (Fig. 10A–D, respectively). Six months after transplantation, a significant decrease was observed in the expression of TNFα by CD56^bright^ NK cells (*P < *0.05) (Fig. 10E). Our findings show that overall there were multiple cell subsets with reduced cytokine expression in the transplant recipients, at 3 and 6 mo post-transplantation.

### Decreased expression of costimulatory molecules by γδ T cells and CD8^+^ T cells in transplant recipients post-transplantation vs baseline

**Figure 11.**
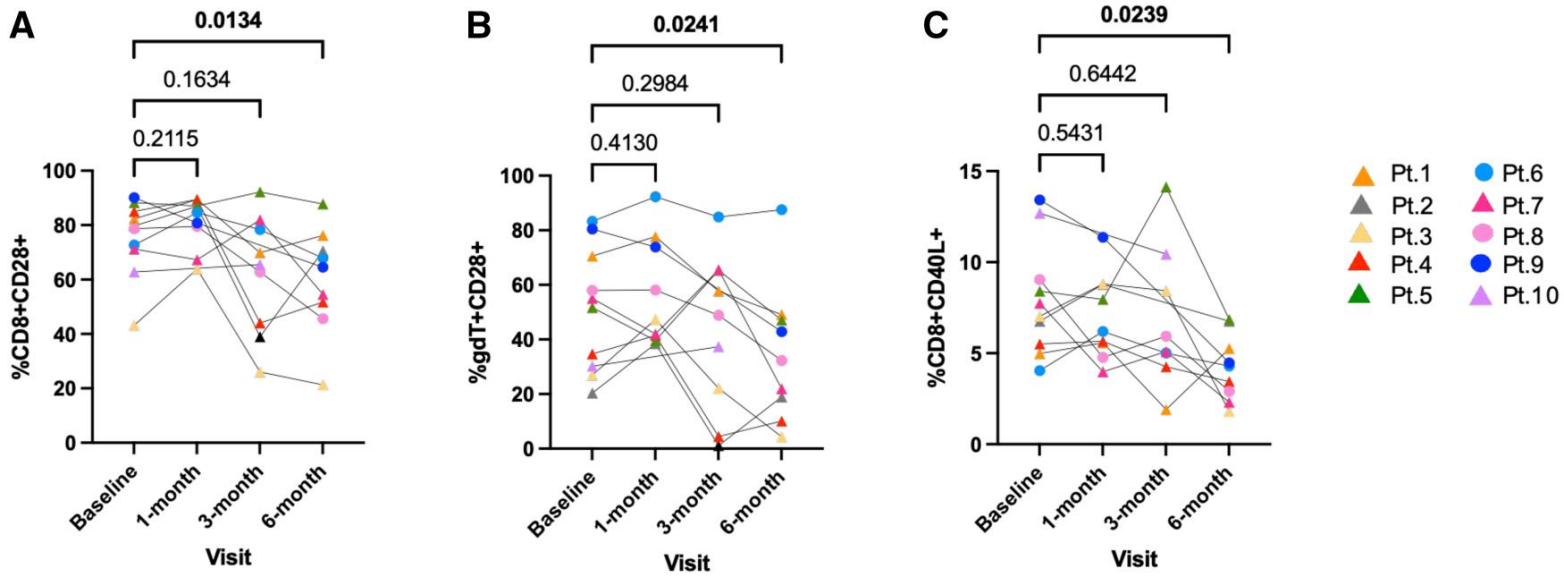
Decreased expression of costimulatory molecules in transplant recipients post-transplantation vs baseline. (A) One-way ANOVA (nonparametric or mixed) showed significant decreased cell frequencies of unstimulated CD28^+^ CD8^+^ T cells at 6 months post-transplant compared to baseline. (B) Similar decreases were seen on unstimulated CD28^+^ γδ T cells. (C) This was also true for PMA + ionomycin stimulated CD40L^+^ CD8^+^ T cells. Data are represented as a percentage of the parent population. For patients, baseline, n = 10; 1 mo, n = 9; 3 mo, n = 8; and 6 mo, n = 9. Graphs show mean ± SEM for each subset. Adjusted *P* values were calculated using Dunnett’s multiple comparisons test. Cohort representation: triangle indicates patients who received rabbit ATG induction therapy; circle indicates patients who did not receive rabbit ATG induction therapy. Pt, patient.

Unstimulated PBMCs showed expression of CD8^+^ CD28^+^ T cells and γδ T CD28^+^ cells to be significantly lower at 6 mo after transplantation in the renal patients compared with their baseline (*P < *0.05) (Fig. 11A, B). Following 4 h PMA + ionomycin stimulation, expression of CD8^+^ CD40L^+^ T cells was significantly lower at 6 mo post-transplantation compared with the baseline (*P < *0.05) (Fig. 11C).

### Decreased expression of lytic molecules, CD107a, and CD57 expression after immunosuppressive therapy in the transplant recipients

**Figure 12.**
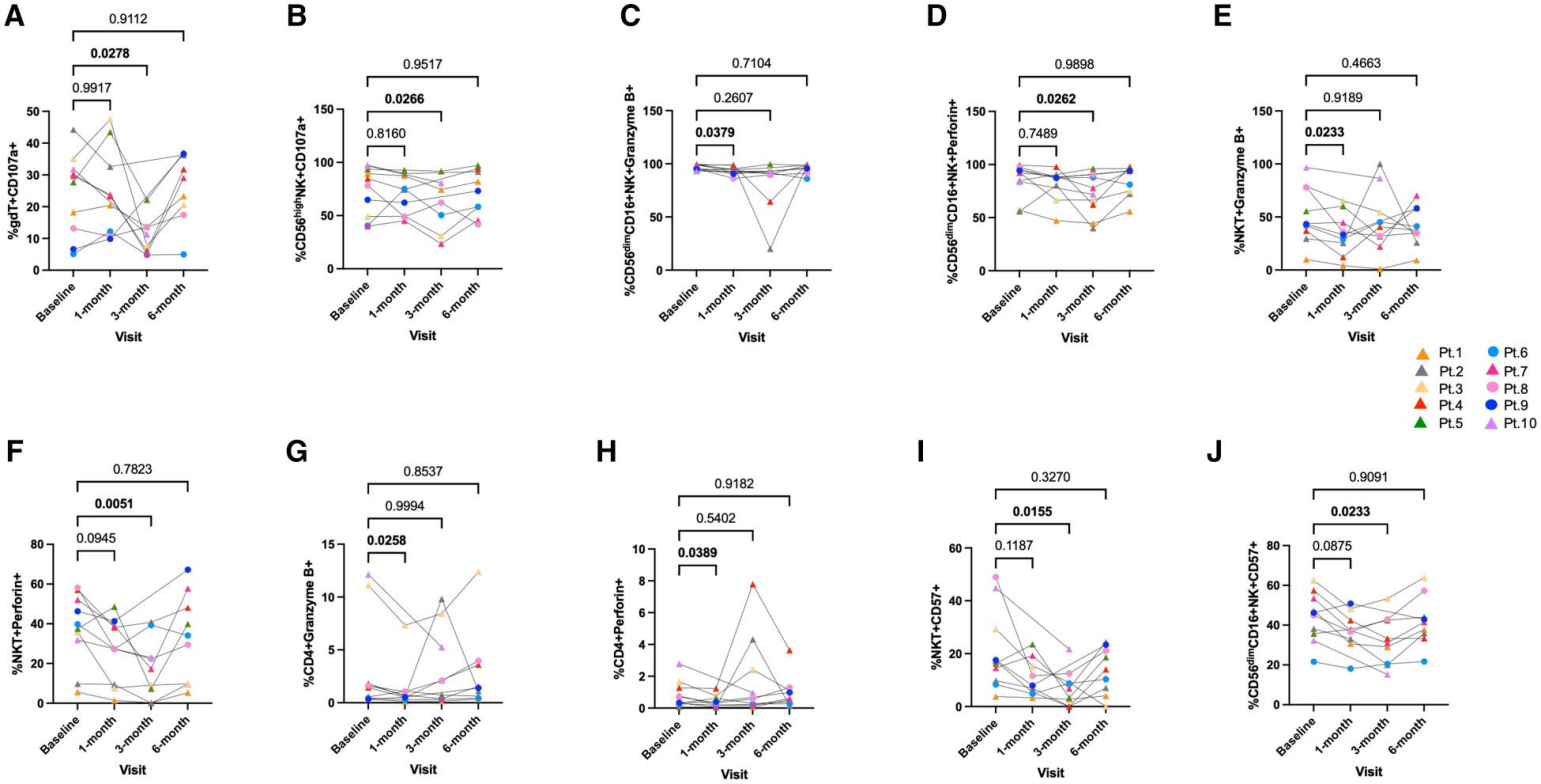
Decreased expression of lytic molecules, CD107a and CD57 expression after immunosuppressive therapy in the transplant recipients. Cell frequencies of CD017a on PMA + ionomycin stimulated (A) γδ T cells and (B) CD56^bright^ NK cells; granzyme B on unstimulated (C) CD56^dim^CD16^+^ NK cells, (E) NKT cells, and (G) CD4+ T cells; perforin on unstimulated (D) CD56^dim^CD16^+^ NK cells, (F) NKT cells, and (H) CD4+ T cells; and CD57 on unstimulated (I) NKT cells and (J) CD56^dim^CD16^+^ NK cells post-transplantation vs baseline. Data are represented as a percentage of the parent population. For patients, baseline, n = 10; 1, 3, and 6 mo, n = 9. Graphs show mean ± SEM for each subset. Adjusted *P* values were calculated by performing 1-way ANOVA (nonparametric or mixed) using Dunnett’s multiple comparisons test. Cohort representation: triangle indicates patients who received rabbit ATG induction therapy; circle indicates patients who did not receive rabbit ATG induction therapy. Pt, patient.

Finally, we examined the immunomodulation of the cytotoxic cells to determine changes after immunosuppressive treatment in the transplant recipients. Along with a decrease in cytokine production observed by γδ T cells at 3 mo post-transplantation (*P < *0.05) (Fig. 10D), we observed a significant reduction in the expression of CD107a by the stimulated γδ T cells (*P < *0.05) (Fig. 12A). CD107a expression was also observed to be significantly decreased by stimulated CD56^bright^ NK cells 3 mo after transplantation compared with the baseline (*P < *0.05) (Fig. 12B). Analysis of the cytotoxic function of unstimulated PBMCs from the transplant recipients showed small but significant differences in granzyme B and perforin expression by CD56^dim^CD16^+^ NK cells at 1 and 3 mo after transplantation, respectively, compared with the baseline (*P < *0.05) (Fig. 12C, D). NKT cells showed a significant decrease of granzyme B expression 1 mo after transplantation (*P < *0.05) and perforin 3 mo after transplantation compared with the baseline (Fig. 12E, F). Interestingly, CD4^+^ T cells and not CD8^+^ T cells showed a decreased expression of both the lytic granules 1 mo after transplantation (*P < *0.05) (Fig. 12G, H).

Further, a significant decrease in CD57 expression on the NKT cells 3 mo after transplantation compared with the baseline was observed in unstimulated PBMCs (*P < *0.05) (Fig. 12I). A significant reduction in CD57 expression on the NKT cells was also found at 3 mo in our analysis between healthy donors and the transplant recipients (*P < *0.05) (Fig. S3, unstimulated). Additionally, the expression of CD57 by the CD56^dim^CD16^+^NK cells was reduced 3 mo post-transplantation vs baseline (*P < *0.05) (Fig. 12J).

## Discussion

In this study, we used a 38 immune marker panel selected to primarily profile T cells along with other immune subsets^10^^,^^20–22^ to comprehensively study immune differences between (1) healthy donors and transplant patients and (2) effects of immunosuppression post-transplantation compared with baseline within the transplant patient cohort. Our comprehensive CyTOF analysis identified CD56^dim^CD16^+^ NK cells to be significantly decreased 1 and 3 mo after renal transplantation compared with healthy donors. Numerous studies have indicated that immunosuppressive treatments significantly impact both the counts and function of NK cells. Our findings confirm studies reporting inhibition of NK cell function with calcineurin inhibitors such as tacrolimus^23^^,^^24^ in which the significant decline in NK cell activity observed in early transplantation patients may be due to the higher doses of the immunosuppressive drugs administered to them compared with those given to patients further post-transplantation. Our results are also consistent with a previous study involving renal transplant recipients, which showed an initial reduction in NK cell counts in patients treated with both tacrolimus and cyclosporin. Nevertheless, NK cell counts were restored in the tacrolimus-treated group 1 yr after transplantation, in contrast to the cyclosporin-treated group.^25^ In our study, we also noted a partial recovery of CD56^dim^CD16^+^ NK cells 6 mo after transplantation. Further, in a study by Morteau et al.,^24^ the authors showed that patients treated with alemtuzumab (reported to preserve NK cells) shortly after transplantation had NK cell counts that were 4 times lower than healthy control subjects. Furthermore, they found that the proportion of CD56^dim^CD16^+^ NK cells was slightly lower, although not significant, in early transplant patients treated with alemtuzumab compared with healthy control subjects. Collectively, our results suggest that conventional immunosuppressive therapies can result in a decrease in both the number and function of NK cells shortly after transplantation at 1 and 3 mo. This could potentially compromise the ability to defend against opportunistic infections and the development of tumors in these patients.

At the phenotypic level, we also found a significant decrease in plasmablasts at all time points after immunosuppressive therapy compared with age-matched healthy donors. Within the transplant cohort, plasmablasts were significantly reduced 1 mo after immunosuppressive therapy with a reduction trend at 3 and 6 mo postimmunosuppression vs baseline. B cells play an important role in renal transplantation in the context of both T cell–mediated and antibody-mediated rejection (AMR).^26^ Two distinct B-lineage cell types have been identified in renal biopsies at the time of acute rejection: CD38^+^ plasmablasts and plasma cells and CD20^+^ B cells. Biopsies from transplants experiencing acute rejection have shown a correlation between CD20^+^ and CD38^+^ immunohistochemistry staining and resistance to steroid therapy.^27^ The presence of CD38^+^ cells, predominantly plasmablasts and plasma cells also correlates with both circulating donor-specific antibody and C4d staining in peritubular capillaries^26^^,^^27^ C4d deposition in renal transplants is believed to stem from antibody-mediated activation, and the presence of C4d in renal biopsies is a hallmark of AMR, as it can cause injury to the renal allograft, leading to reduced graft function and potentially graft loss. The CD38^+^ cells may therefore represent the progenitor pool for long-lived plasma cells that produce antibodies, contributing to AMR. CD38 staining has also been proposed as an alternative immunohistochemistry marker for detecting AMR in renal recipients.^26–28^ Further, as noted by N. Kambham et al. (unpublished data), the clinical significance of both CD20 and CD38 markers is relevant specifically in cases of acute graft rejection, as these markers appear in infiltrates linked to subcapsular scarring, reflux nephropathy, and acute pyelonephritis. In our patient cohort, 2 patients (patient 7 and patient 4) showed elevated plasmablast levels at 6 mo and rejected their transplant. Patient 7 had a T cell–mediated rejection 6 mo after transplantation. For patient 4, the clinical report showed that the patient had calcineurin toxicity and was therefore removed from Prograf and received only mycophenolate mofetil at 6 mo after transplantation. This patient also appeared to have low level BK viremia and *Escherichia coli* urinary tract infection. CyTOF analysis revealed elevated levels of CD4^+^ CD38^+^HLA-DR^+^ and CD8^+^ CD38^+^HLA-DR^+^ cells in this patient, suggesting a biologically activated immune state. Additionally, increased levels of both proinflammatory and regulatory cytokines were observed in their immune profile. Previously, a study by Mota et al.^29^ identified a prevalence of regulatory cytokines, with IL-10 being a prominent finding within 2 yr post-renal transplantation. Similarly, we detected increased IL-10 levels in CD4^+^, CD8^+^, and γδ T cells from PBMCs in this patient by CyTOF. Furthermore, some studies have also indicated that the suppression of proinflammatory cytokines like IL-2 may correlate with short-term graft tolerance. Conversely, increased levels of proinflammatory plasma cytokines might be associated with a deterioration in renal function, especially in the long term.^29–31^ We found elevated levels of IL-2^+^ CD4^+^ T cells in this patient. We therefore speculate that patient #4 could have had an AMR and/or a T cell–mediated rejection.

Furthermore, our CyTOF analysis revealed no differences in the relative percentages of total T cells, in the T cell subtypes and B cells, between patients’ pretreatment (baseline) and healthy donors, consistent with findings reported in another study by Wang et al.^32^ This suggests that the lymphocyte composition remained unaffected by the underlying disease that led to the transplantation. Similar to the findings from Wang et al., our analyses also identified a significantly lower percentage of naïve CD8^+^ T cells 6 mo after transplantation compared with the healthy donors. Additionally, a significant increase was observed in the percentage of CD8^+^ effector memory T cells re-expressing CD45RA (TEMRA) compared with both healthy donors and the pretransplant baseline in patients 6 mo following transplantation. These findings suggest that post-transplantation the T cell memory subsets shift toward a more terminally differentiated phenotype.^32^ The inclusion of older patients (>60 yr) in our cohort may have contributed to a decrease in the frequency of naïve CD8 T cells. This decline potentially explains an impaired immune system's ability to respond to new pathogens, as there is a limited pool of naïve T cells available for activation and differentiation. Furthermore, the observed increase in the frequency of the TEMRA subset could suggest an impaired ability of memory T cells to proliferate, contributing to a partial loss of immune responses to previously encountered antigens.^33^ Further, when comparing patients pre- and post-transplantation we found significant differences in the relative frequencies of the T cell subsets and the B cell subsets. There was a significant decrease in CD4^+^ T cells 3 mo after initiating immunosuppressive therapy, followed by a partial recovery of CD4^+^ T cells that was not statistically significant at 6 mo compared with baseline. These findings align with other studies that have reported significant ATG-induced CD4 lymphopenia in the first year following renal transplantation, linking it to an increased risk of infections and cancer.^34–39^ Additionally, we found a significant increase in DN (CD3^+^CD4^−^CD8^−^) T cells at 1 and 3 mo post-transplantation. Another research study published recently also drew a correlation between increased DN (CD3^+^CD4^−^CD8^−^) T cells and stable renal graft function in the first year.^40^^,^^41^ B cell immune response following transplantation can engage various B cell populations.^42–45^ Previous studies have indicated that B cells can contribute to both antibody-dependent and antibody-independent responses in the context of transplant tolerance or rejection. However, there are limited data available to fully comprehend the distribution and function of B cell subsets in renal transplant recipients.^46^ In this study, we observed a notable increase in the relative proportions of B cells at 1 mo post-transplantation compared with pretransplant levels, suggesting an activated B cell response shortly after transplantation. Subsequently, there was a decrease, although not statistically significant, at 3 and 6 mo post-immunosuppressive therapy. However, our panel lacked markers to thoroughly examine the various B cell subsets and their functions in the context of renal transplantation, warranting the need for further investigation.

Our T cell phenotypic findings are supported by functional immune data revealing an alignment between cytokine and costimulatory profiles and the maintenance of stable graft function. Previous studies on gene expression have demonstrated heightened levels of IL-2 and IL-4 gene expression, alongside diminished IL-10 expression, in renal prerejection samples compared with control and post-transplantation samples.^47^ In our current study, within the transplant cohort, we observed reduced expression of IL-2 and IL-4 by T cells 3 mo after transplantation. Additionally in our comparative analysis between transplant patients and healthy donors, we found decreased expression of TNFα, IFNγ, MIP-1β and GM-CSF by γδ T cells and CD8 T memory cell subsets. Research in transplantation has indicated that T cells secrete both proinflammatory and T helper 2/regulatory cytokines in response to transplants, with TNFα identified as a critical mediator in graft rejection.^48–52^ Thus, our results indicate the presence of immunosuppression of T cell–mediated immune responses. Additionally, our CyTOF data illustrated a decrease in the expression levels of the costimulatory molecules CD28 and CD40L by the CD8 and γδ T cells in transplant recipients compared with baseline transplant levels and those of healthy donors. Costimulatory signals are essential for initiating T cell immune responses, and the inhibition of costimulatory factors has been linked with lack of graft rejection.^53^^,^^54^

A prime mechanism in solid organ transplant rejection involves the activation of granzymes and perforin by cytotoxic T lymphocytes and NK cells.^13^^,^^55^^,^^56^ Our CyTOF analysis also revealed changes in the phenotype of lytic granules following transplantation. While we saw a decrease in the expression of granzyme B and perforin at 1 and 3 mo by the NKT, γδ T, CD4^+^ T cells, and CD56^dim^CD16^+^ NK cells in the patients compared with their baseline samples, we also found an increase in the expression of granzyme B by the CD8^+^ T cells and CD56^bright^NK cells in the patients post-transplantation compared with the healthy donors. A study examining perforin expression in the peripheral blood of tolerant kidney recipients reported patients who maintained renal transplants for an extended period had very low levels of CD4^+^ perforin and CD8^+^ perforin expression.^57^ While no significance in the CD8^+^ perforin expression was reached in our cohort, 1 mo post-transplantation the percentage of CD4^+^ perforin was <1% compared with pretransplant. Additionally, 6 mo post-transplantation, we observed an increase in granzyme B expression by the CD8 T cells. This implies that there is an initially subdued immune response promoting graft tolerance right after transplantation, which is succeeded by an activated immune response in the early stages of transplantation, even with ongoing immune suppression treatment. Evidence of an activated immune system 6 mo post-transplantation was further supported by the presence of rapid inflammatory responses, characterized by both the elevation of cytotoxic function (CD107a) and the secretion of proinflammatory cytokines IFNγ and MIP-1β by CD8^+^ T cells.^58^ Furthermore, it is worth noting that deficient CD8^+^ CD107a production has been associated with cytomegalovirus infection. Considering that the transplant recipients in this group were administered valganciclovir, it is plausible to speculate that the elevated levels of CD8^+^ CD107a could be attributed to valganciclovir.^59^ However, despite the indication of an activated immune system, as evidenced also by the heightened expression of CD57 by CD8^+^ T cells, recent research has revealed that a higher proportion of senescent CD8^+^CD57^+^ T cells in renal transplant patients^33^ undergoing immunosuppressive treatment with tacrolimus (FK506) experienced improved graft acceptance.^60^ This is further supported by the decreased expression of CD57 by the NK cells within the transplant cohort.^61^ Consistent with this interpretation of graft tolerance was an inhibition of NK cell degranulation.^24^

In conclusion, the findings of our longitudinal study, prestudy, and post-transplantation time points unveiled the multifaceted nature of the immune profile in renal transplant patients. In general, our data reveal a suppression of the immune system consistent with the ongoing immunosuppressive regimen. Moreover, 6 mo after transplantation, we also noted an immune profile suggesting activation despite patients continuing to receive immunosuppressive therapy. Our study had limitations, including the relatively small sample size and the absence of additional time points. Additionally, the analysis is based on relative frequency. However, it is worth noting that the cohort size is consistent with previous successful CyTOF studies in various fields, aligning with established practices.^62–65^ Collectively, our findings underscore the potential of CyTOF as a potent technology capable of facilitating comprehensive profiling of immune components. This capability, in turn, allows for the prediction of responses to treatments, thus paving the way for more informed and optimized individual treatment strategies. Additionally, it highlights the necessity for developing improved immunosuppressants that specifically target the hyperactivated immune components to promote favorable long-term outcomes following renal transplantation.

## Supplementary Material

vlae013_Supplementary_Data

## Data Availability

Data available upon request.
