# Supplementary material for: Characterization of immune phenotypes in peripheral blood of adult renal transplant recipients using mass cytometry (CyTOF)
Source: Immunohorizons. 2025 Feb 18;9(4):vlae013. doi: 10.1093/immhor/vlae013 (PMC11841977; doi:10.1093/immhor/vlae013)
Supplement: vlae013_Supplementary_Data [file vlae013_supplementary_data.pdf]

SUPPLEMENTARY TABLE:

Table S1. Detailed demographic features of the patient population

	Patient _01	Patient_02	Patient _03	Patient_04	Patient_05	Patient_06	Patient_07	Patient_08	Patient_09	Patient_10
**Gender**	M	M	M	M	F	F	M	M	F	F
**Age at Transplant**	37 y.o.	26 y.o.	43 y.o.	40 y.o.	56 y.o.	40 y.o.	31 y.o.	47 y.o.	71 y.o.	56 y.o.
**Primary Disease**	End stage renal disease secondary to Alport Syndrome	End stage renal disease secondary to Focal Glomerular Sclerosis	Stage IV Chronic Kidney Disease secondary to autosomal dominant polycystic kidney disease	End stage renal disease secondary to Chronic Glomerulosclerosis, unspecified	End stage renal disease secondary to lupus nephritis	End stage renal disease secondary to IgA Nephrophathy	End stage renal disease secondary to congenital obstructive uropathy	End-stage renal disease secondary to IgA nephropathy	End-stage renal disease secondary to oxalate nephropathy	End-stage renal disease secondary to nephrectomy required for von Hippel-Lindau syndrome and Renal Cell Carcinoma
**Induction IS/Date**	rATG, Methylprednisolone	rATG, Methylprednisolone	rATG, Methylprednisolone	rATG, Methylprednisolone	rATG, Methylprednisolone	Methylprednisolone	rATG, Methylprednisolone	Methylprednisolone	Methylprednisolone	rATG (Methylprednisolone started 2 days after transplant)
**Viral Prophylaxis/Date**	Valganciclovir (Valcyte)	Valganciclovir (Valcyte)	Valganciclovir (Valcyte)	Valganciclovir (Valcyte)	Valganciclovir (Valcyte)	Valganciclovir (Valcyte)	Valganciclovir (Valcyte)	Valganciclovir (Valcyte)	Valganciclovir (Valcyte)	None - Valganciclovir (Valcyte) started 2 days after transplant
**Maintenance drugs/dose/levels**	Prograf 3 mg PO BID, Cellcept 100 mg PO BID	Prograf 2 mg PO BID, Cellcept1g IV q12	Prograf 2 mg PO BID, Cellcept1g IV q12	Prograf 1 mg PO BID, Cellcept1g IV q12	Prograf 1.5 mg PO BID, Cellcept 100 mg PO BID	Prograf 2 mg PO BID, Cellcept 100 mg TID	Prograf 2 mg PO BID, Cellcept1g IV q12	Cellcept 100 mg TID	Prograf 2mg PO BID, Cellcept 500 mg PO TID	Prograf 2 mg PO BID, Cellcept 1 g PO q12
**EBV serology (R)**	6/13/17: VCA IgG 7.37 (positive), VCA IgM negative, EA IgG negative, EBNA IgG 2.7 (positive)	9/5/17: VCA IgG 2.92 (positive), VCA IgM negative, EA IgG negative, EBNA IgG 2.36 (positive)	9/18/17: VCA IgG 6.85 (positive), VCA IgM negative, EA IgG negative, EBNA IgG 2.80 (positive)	10/3/17: VCA IgG negative, VCA IgM negative, EA IgG negative, EBNA IgG 1.71 (positive)	10/31/17: VCA IgG 11.25 (positive), VCA IgM negative, EA IgG 3.07 (positive), EBNA negative	11/8/17: VCA IgG 10.17 (positive), VCA IgM negative, EA IgG negative, EBNA 2.77 (positive)	11/14/17: VCA IgG 8.26 (positive), VCA IgM negative, EA IgG negative, EBNA IgG 3.62 (positive)	2/6/18: VCA IgG 6.06 (positive), VCA IgM negative, EA IgG negative, EBNA IgG 2.67 (positive)	2/13/18: VCA IgG 8.38 (positive), VCA IgM negative, EA IgG negative, EBNA IgG 2.91 (positive)	3/20/18: VCA IgG 10.08 (positive), VCA IgM negative, EA IgG negative, EBNA IgG 2.54 (positive)
**EBV serology (D)**	3/8/17: VCA IFF 7.73 (positive)	5/10/17: VCA IgG 2.79 (positive), VCA IgM not done, EA IgG not done, EBNA IgG not done	12/21/16: VCA IgG 6.67 (positive)	9/18/17: VCA IGG Negative	4/19/17: VCA IGG 7.28 (positive)	6/21/17: VCA IGG 10.94 (positive)	8/15/17/17: VCA IGG 8.82 (positive); 8/8/17 EA IgG 34.2 (positive)	10/18/17: VCA IGG 1.31 (positive)	9/6/17: VCA IGG 3.79 (positive)	10/24/17: VCA IGG 4.24 (positive)
**CMV serology (R)**	6/13/17: CMV IgG negative	9/5/17: CMV IgG 54 UA/ML (positive)	9/18/17: CMV IgG 39 UA/ML (positive)	9/24/15: CMV IgM 1.5 (positive), CMV IgG >5 (positive) - Quest	10/31/17:CMV igG>400 UA/ML (positive)	11/8/17: CMV IgG 47 UA/ml (positive)	11/14/17: CMV IgG negative	1/30/18: CMV IgG 30 UA/ml(positive)	2/13/18: CMV IgG 42 UA/ml (positive)	3/20/18: CMV IgG 29 UA/ml (positive)
**CMV serology (D)**	6/14/17: CMV IgG negative	5/10/17: CMV IgG 103 UA/ML (positive)	12/21/16: CMV IgG 61 UA/ML (positive)	9/18/17: CMV IgG 104 UA/ML (positive)	4/19/17: CMV IgG negative	6/21/17: CMV IgG 9 UA/ML (positive)	11/17/17: CMV IgG negative	10/18/17: CMV IgG 31 UA/ML (positive)	9/6/17: CMV IgG negative	10/24/17: CMV IgG 47 UA/ML (positive)
**CMV viral load/date**	No data found	No data found	No data found	1/13/18; Plasma, not detected; 3/1/8: Plasma, detected (316 IU/ML); 3/8/18: Plasma detected (315 IU/ML); 3/28/18: Plasma, detected (<135 IU/ML; 7/2/18: Plasma, not detected	1/26/18: Plasma, Not detected	No data found	No data found	No data found	No data found	No data found
**EBV viral load/ date**	No data found	No data found	No data found	1/13/18 nd 3/28/18: Plasma, Not detected;	5/7/18 : Plasma, Not Detected	No data found	No data found	No data found	No data found	No data found
**Followup after 6 month draw**				transplant rejection 10/18			12/18/18 Borderline T cell mediated rejection; treated with pulse of oral steroids for 3 days			
**Other**		preformed low level donor specific antibody to the antigen DR52 with an MFI of 1066, been maintained on prednisone due to concerns about his low-level DSA to DR52; 1 yr post-transplant: Ulcerative colitis/proctitis; 4 months posttransplant: erythrocytosis; anemia		3 months post transplant low level BK viremia; E coli UTI; calcineurin inhibitor toxicity						
**IS month 3**	Prograf 5 mg PO BID, Cellcept 500 mg PO BID, Prednisone 5 mg OD	Prograf 1 mg PO AM + 1.5 mg PO PM, Cellcept 250 mg PO BID, Prednisone 5 mg OD	Prograf 1 mg PO BID, Cellcept 750 mg PO BID, Prednisone 4 mg OD	Tacrolimus (Envarsus XR) 2 mg PO OD, Cellcept 500 mg PO BID, Prednisone 5 mg OD	Prograf 2 mg PO BID, Cellcept 500 mg PO BID, Prednisone 5 mg OD	Prograf 5 mg PO BID, Cellcept 750 mg PO BID, Prednisone 5 mg OD	Prograf 2 mg PO AM and 3 mg PO PM, Cellcept 1000 mg PO BID, Prednisone 5 mg OD	Prograf 1.5 mg BID, Cellcept 750 mg PO BID, Prednisone 5 mg OD	Prograf 1 mg PO AM and 1.5 mg PO PM, Cellcept 500 mg PO BID, Prednisone 5 mg OD	Prograf 2 mg PO AM and 3 mg PO PM, Cellcept 500 mg PO BID, Prednisone 5 mg OD
**IS month 6**	Prograf 4 mg PO BID, Cellcept 500 mg PO BID, Prednison 5 mg OD	Prograf 1 mg PO AM + 1.5 mg PO PM, Cellcept 500 mg PO BID, Prednisone 5 mg OD	Prograf 1 mg PO BID (except for M-W-F when he takes 0.5 mg AM and 1 mg PM), Cellcept 750 mg PO BID, Prednisone on taper, currently 1 mg OD	Cellcept 250 mg PO OD	Prograf 2 mg PO BID, Cellcept 250 mg PO BID, Prednisone 7.5 mg OD	Prograf 4 mg PO AM AND 5 MG PO PM, Cellcept 500 mg PO BID, Prednisone 5 mg OD	Prograf 3 mg PO BID, Cellcept 750 mg PO BID, Prednisone 5 mg OD	Prograf 1 mg BID, Cellcept 750 mg PO BID, Prednisone 5 mg OD	Prograf 1 mg PO BID, Cellcept 500 mg PO BID, Prednisone 4 mg OD	No change

SUPPLEMENTARY FIGURES:

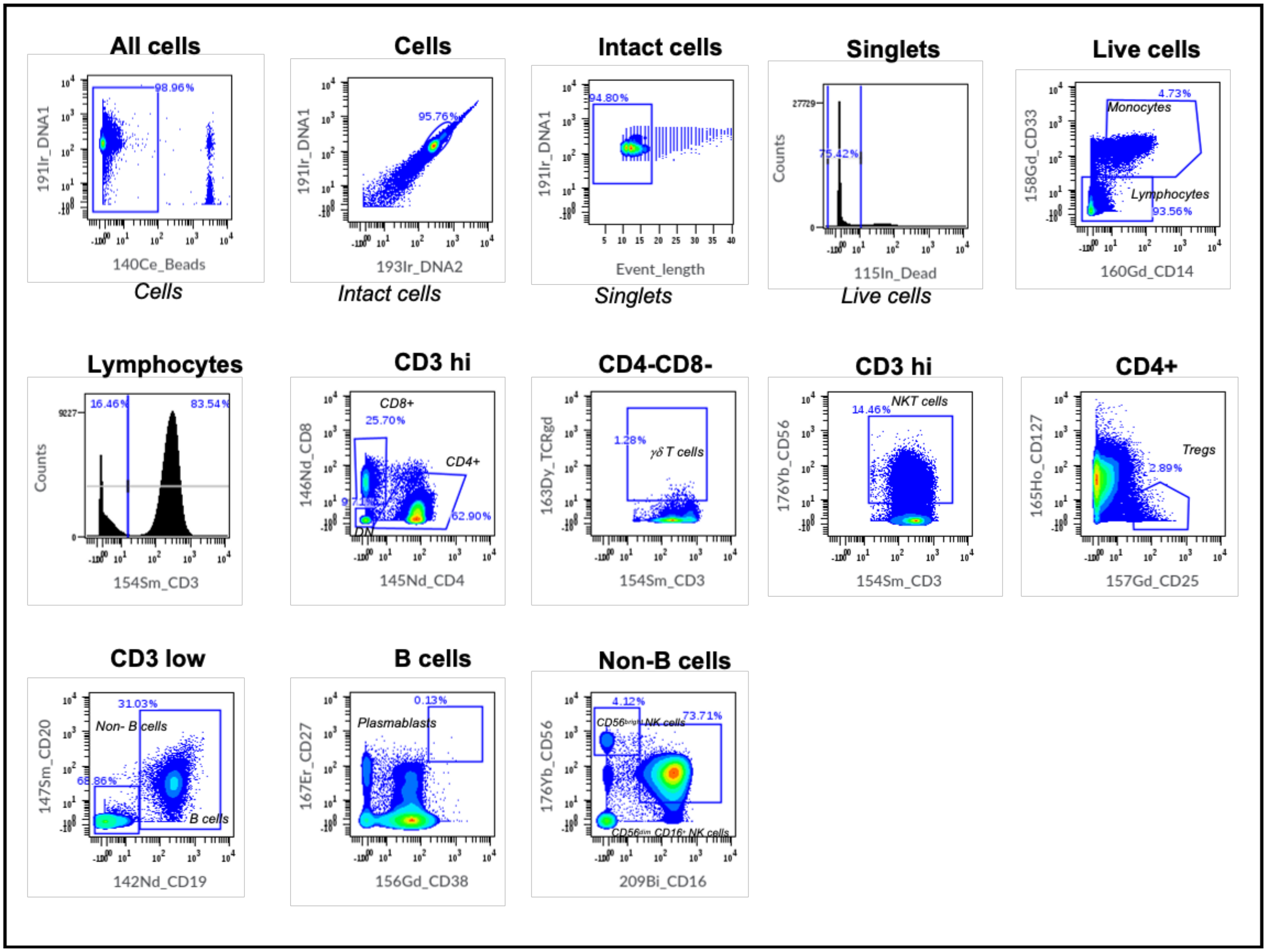

Figure S1. CyTOF Gating Strategy Peripheral Blood Mononuclear Cells (PBMCs). Post normalization using the MATLAB normalizer, normalized .fcs files were uploaded in Cytobank (www.cytobank.org). Major immune cell subsets from the PBMCs were gated following a manual gating strategy. First cells were gated to exclude the normalization beads, following which we gated on intact cells to exclude the debris. Next, we gated on the singlets and subsequently the live cells to eliminate the dead cells. From the live cells, we used a hierarchical gating strategy to identify the main lineage immune populations including monocytes, lymphocytes, T cells, $CD4^+$ and $CD8^+$ T cells, NKT cells, gd T cells, Tregs, B cells and Natural Killer (NK) cells.

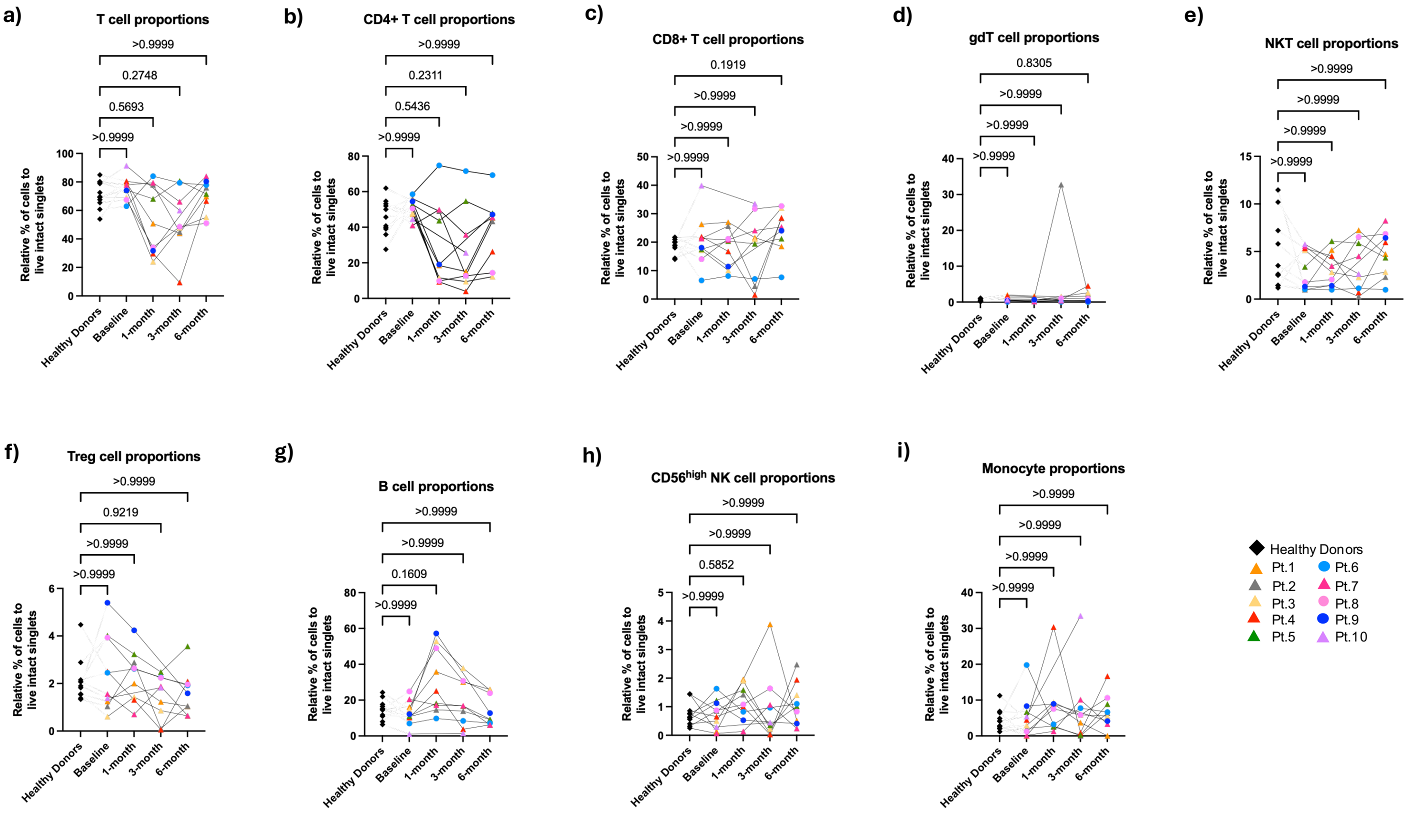

Figure S2. Phenotypic expression of major immune subsets. Cell frequencies of unstimulated PBMCs from healthy donors (average of baseline, 3- and 6-months) and renal transplant recipients across all time-points is shown (A) T cells (B) $CD4^+$ T cells (C) $CD8^+$ T cells (D) γδ T cells (E) NKT cells (F) Tregs (G) B cells (H) $CD56^{bright}$ NK cells (I) Monocytes. Data is normalized to live intact singlets. For healthy donors n=11. For patients: baseline, n = 10; 1 month, n = 9; 3 months, n = 8 and 6 months, n = 9. Graphs show mean ± SEM for each subset. One-Way ANOVA was performed using the Dunn's multiple comparisons test. Cohort representation- diamond: healthy donors; triangle: patients who received rATG induction therapy; circle: patients who did not receive rATG induction therapy.

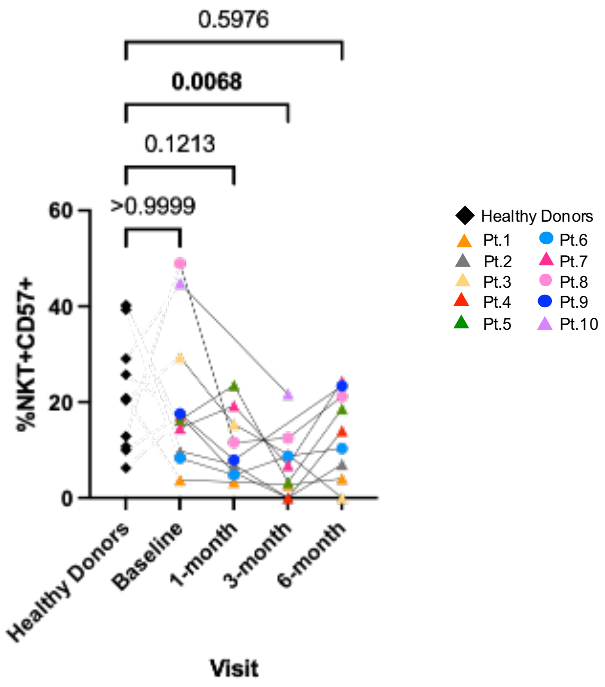

Figure S3. CD57 expression on NKT cells. One-Way ANOVA showed that unstimulated PBMCs of renal transplant recipients had significant decreased expression of CD57 in NKT cells 3-months after transplantation compared to healthy donors. Data is represented as a percentage of the parent population. For patients, baseline, n = 10; 1 month, n = 9; 3 months, n = 8 and 6 months, n = 9. For healthy controls n = 11 (average of baseline, 3- and 6- months). Graphs show mean ± SEM for each subset. Adjusted p-values were calculated by one-way ANOVA using the Dunn's multiple comparisons test. **p < 0.01. Cohort representation- diamond: healthy donors; triangle: patients who received rATG induction therapy; circle: patients who did not receive rATG induction therapy.
